# The patterns of microbial community distribution and co-occurrence in water columns and sediments of Haima cold seep

**DOI:** 10.1128/spectrum.00256-26

**Published:** 2026-06-11

**Authors:** Changyu Zhu, Tong Wei, Yuxuan Lin, Guoyong Yan, Pei-Yuan Qian

**Affiliations:** 1 Southern Marine Science and Engineering Guangdong Laboratory (Guangzhou) 606379 , Guangzhou, China; 2 Department of Ocean Science, The Hong Kong University of Science and Technology, Hong Kong, China; 3 Otto Poon Center for Climate Resilience and Sustainability, The Hong Kong University of Science and Technology, Hong Kong, China; Ocean University of China, Qingdao, Shandong, China

**Keywords:** cold seep, microorganism, distribution pattern, co-occurrence pattern, ecological process

## Abstract

Deep-sea cold seeps are essential components of global biodiversity and biogeochemical cycles, whereas the community distribution and co-occurrence patterns of cold seep microorganisms remain poorly understood. Here, we investigated the microeukaryotic and prokaryotic communities across different water layers and benthic habitats in the Haima cold seep of the South China Sea and revealed distinct vertical and horizontal patterns in microbial distribution and co-occurrence between the water columns and sediments. Prokaryotic intra-domain interactions predominated over inter-species correlations in networks, and bacteria–microeukaryote interactions were more abundant in the network of water columns than in sediments, indicating that environmental preferences of microorganisms differed between these environments. The environmental factors and deterministic processes appeared to exert a more pronounced influence on the distribution patterns of microeukaryotes than on prokaryotes in the water columns, whereas the opposite was observed in the sediments. Additionally, microbial co-occurrence patterns were segregated and primarily shaped by deterministic processes. The species interactions were more complex in the euphotic zone and clam (one of the most dominant megabenthos) habitats than those in other water layers and benthic habitats, and the microbial co-occurrence patterns in the euphotic zone and mussel beds were likely influenced by strong selection factors such as light, organic carbon, mussel activities, and high methane concentrations. Our study provides new perspectives on how microorganisms adapt to the different environmental conditions in deep-sea cold seeps, deepening our understanding of microbial ecology in deep-sea extreme environments.

**IMPORTANCE:**

Our study revealed the vertical and horizontal patterns of microbial community distribution and co-occurrence, highlighting how different ecological processes shape microbial communities in the water column and sediments, as well as between microeukaryotes and prokaryotes. This indicated that the balance of deterministic and stochastic processes was influenced by the environment and taxonomic classification in deep-sea cold seeps. The species interactions in the euphotic zone and clam habitat were more complex than those in other water layers and benthic habitats, and the euphotic zone and mussel beds served as hotspots of intense environmental selection. Our findings revealed that microorganisms responded to these challenging environments in deep-sea cold seeps through various strategies, including different environmental preferences. By elucidating the mechanisms driving the microbial community diversity and species coexistence, this work provides new perspectives on how microorganisms adapt to the environments in deep-sea cold seeps.

## INTRODUCTION

Cold seeps are unique deep-sea ecosystems characterized by the continuous or intermittent discharge of methane-, sulfide-, and hydrocarbon-rich fluids from subsurface geological reservoirs into the surrounding seafloor environment (1, 2). Such seepage processes not only shape distinct seafloor geomorphology but also support diverse microbial communities with specialized metabolic functions (3, 4). Given their capacity to support high biomass and diverse life forms in the generally oligotrophic deep-sea environment, cold seeps are often described as “oases in the deep-sea desert” (5, 6). Furthermore, seepage processes can markedly enhance the productivity of the overlying water column (WC), mitigate environmental limitations, and promote rich microbial diversity with unique community assemblages (7, 8). Microbial communities, as essential components of cold seep ecosystems, play crucial roles in facilitating biogeochemical cycling and energy flow through key processes, such as anaerobic oxidation of methane and sulfate reduction (9, 10). Thus, a systematic investigation of microbial diversity and distribution patterns in cold seep sediments and different water layers is essential for understanding the structure and function of cold seep ecosystems. Notably, deep-sea cold seeps contain abundant methane hydrate deposits and serve as critical indicators for the environmentally sustainable exploitation of these energy reserves (11–13).

Despite substantial advances in characterizing microbial diversity in cold seeps, current research remains disproportionately focused on specific functional groups (14–19). By contrast, comprehensive investigations that simultaneously analyze prokaryotic and microeukaryotic communities in sediments and the overlying water column remain scarce. Previous studies have been limited to a single habitat (sediments or overlying water columns) or a single microbial domain (prokaryotes or microeukaryotes), yielding a fragmented and incomplete understanding of cold seep microorganisms, particularly for microeukaryotes in water columns (20–22). The complex interactions within microbial communities remain insufficiently resolved, particularly among different domains, such as the interactions between bacteria and microeukaryotes in cold seep ecosystems (23, 24). The principal environmental factors and ecological processes that drive the microbial distribution and co-occurrence in the water columns and benthic habitats of cold seeps are poorly characterized, impeding a comprehensive understanding of microbial diversity and species coexistence (25, 26).

The Haima cold seep, located in the northwestern South China Sea, is one of the largest and most active cold seep systems (27). This cold seep field, situated at a water depth of approximately 1,300–1,400 m, hosts various unique benthic habitats, such as mussel beds, sea anemones, non-fauna seabeds, and dead and live-clam beds, providing a natural laboratory for elucidating microbial distribution, species interactions, and associated ecological processes in the deep sea (28, 29). Previous studies have revealed diverse prokaryotic and microeukaryotic communities in the Haima cold seep, where sulfate-reducing bacteria (SRB) and anaerobic methanotrophic archaea (ANME) play pivotal roles in facilitating local carbon and sulfur cycling (30–32). Depth-stratified investigations in water columns have revealed pronounced changes in dominant prokaryotic taxa, with community diversity exhibiting clear variations along vertical hydrochemical gradients (7). Notably, the assembly of prokaryotic and microeukaryotic communities can be influenced by differing ecological processes (26, 33), and the intensity of seepage may exert varying effects on microbial network complexity (23, 25). These notable discrepancies may reflect strong environmental heterogeneity within cold seep ecosystems, resulting in considerable variations in microbial community assemblages and species coexistence (34). Moreover, habitat-specific conditions, such as mussel beds, sea anemones, non-fauna seabeds, and dead clams and clam beds, may further enhance the divergence of microbial communities across various ecological niches. However, the distribution patterns of microeukaryotes and microbial interactions, especially the cross-domain correlations among different water layers and benthic habitats, remain unknown, which hinders a comprehensive understanding of cold seep ecosystems.

To address these critical knowledge gaps, we conducted an integrated investigation of prokaryotic and microeukaryotic microbial communities in the sediment and overlying water columns of Haima cold seep, using high-throughput sequencing, specifically aiming to (i) characterize the community compositions and distribution patterns of prokaryotes and microeukaryotes in water columns and sediments; (ii) explore the co-occurrence patterns across different microbial domains; and (iii) elucidate the ecological processes that determine the structure of microbial distribution and co-occurrence patterns.

## MATERIALS AND METHODS

### Sample collection and measurement of environmental factors

**Fig 1:**
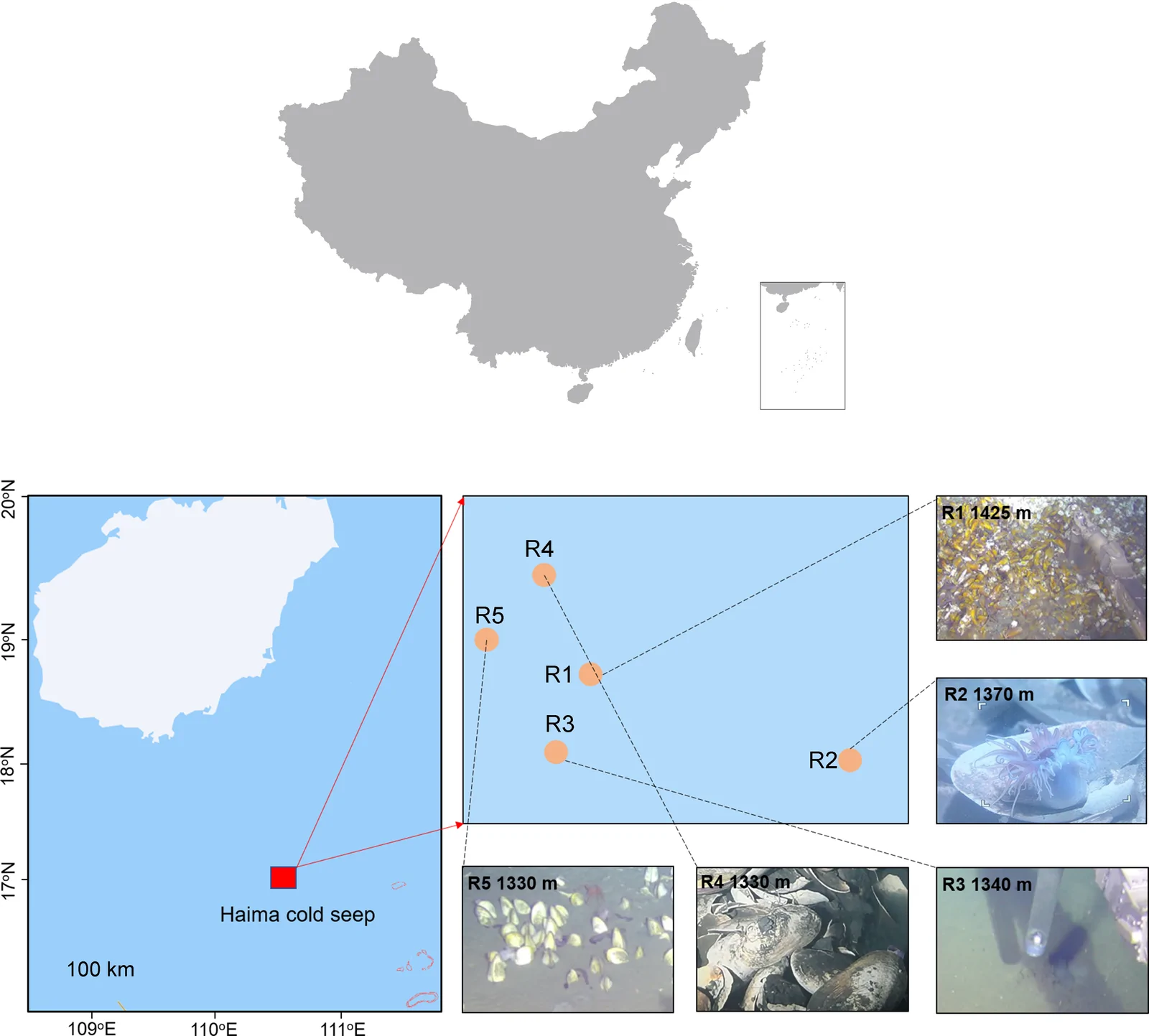
Location of the five sampling sites in the Haima cold seep.

Sampling of the Haima cold seep (16°43′N, 110° 28′E) in the South China Sea was conducted in May 2023 during the HYDZ6-202302 cruise. Five sampling sites were named R1, R2, R3, R4, and R5, each representing different habitats: mussel, sea anemone, non-fauna seabed, dead clam, and clam, respectively (Fig. 1). WCs were collected from epipelagic (0–200 m depth, including 5, 100, and 200 m), mesopelagic (200–1,000 m, including 600 and 1,000 m), and bathypelagic (1,000–4,000 m, including 1,000 m from the surface and at 100, 50, 30, and 10 m from the bottom) layers according to previous studies (35, 36), using rosette Niskin bottles equipped with Conductivity-Temperature-Depth (CTD) instruments. Sediment (SE) samples were collected by push cores using the remotely operated vehicle *Haima 2* at five sampling sites, with subsections at depths of 2, 4, 6, 8, 10, 15, 20, 25, and 30 cm for each core sample. Porewaters were extracted from these nine sediment subsections using Rhizon samplers (Rhizosphere Research Products, Wageningen, the Netherlands) at 4°C in a cold room to measure environmental data. After porewater collection, all sediment samples were maintained at −80°C on board, transferred to the laboratory in dry ice, and stored at −80°C for further analysis.

The depth, temperature (Temp), pH, salinity (Sal), and dissolved oxygen (DO) of the water column samples were measured *in situ* using CTD. The concentrations of nitrite nitrogen (NO_2_^−^), nitrate nitrogen (NO_3_^−^), ammonium nitrogen (NH_4_^+^), phosphate phosphorus (PO_4_^3−^), and dissolved silicate (DSi) in water columns and porewaters were measured using an automatic nutrient analyzer (LACHAT QUITCHEM 8500 S2, HACH, USA). Dissolved organic carbon (DOC) was measured using a TOC analyzer (OI Analytical, USA). For each water column sample, about 8 L of water was *in situ* collected and filtered through a 0.22 μm microporous filter membrane (Supor−200 Membrane Filter, PALL, USA) with a vacuum pump to enrich the prokaryotes and microeukaryotes. Those filters were immediately stored at −80°C on board, transferred to the laboratory in dry ice, and maintained at −80°C for further analysis. A total of 44 water column samples and 45 sediment samples were collected, except for the water column sample at 1,000 m at R1 (WCR1_5) due to a malfunction of the CTD instrument (Table S1).

### DNA extraction and sequencing

Genomic DNA was extracted from WC and SE using the PureLink Genomic DNA kit (Invitrogen, Carlsbad, USA) according to the manufacturer’s instructions. The DNA quality was assessed using a NanoDrop 2000 spectrophotometer (Thermo, ND2000c, USA). The extracted DNA was subsequently used as a template for polymerase chain reaction (PCR) amplification targeting the V4 region of the 16S rDNA for prokaryotes and the 18S rDNA for microeukaryotes. Each 25 μL PCR contained 12.5 μL of Q5 Hot Start High-Fidelity 2× Master Mix (New England Biolabs, MA, USA), 10.5 μL of deionized water, 0.5 μL of each forward and reverse primer, and 1 μL of DNA template. For the amplification of the prokaryotic 16S rDNA V4 region, barcoded primers 515F 5′-GTGYCAGCMGCCGCGGTAA-3′ and 806R 5′-GGACTACNVGGGTWTCTAAT-3′ (37) were used. The PCR amplification procedure was as follows: initial predenaturation at 98°C for 30 s; 30 cycles of 98°C for 10 s, 53°C for 30 s, and 72°C for 1 min; and final extension at 72°C for 10 min. For the 18S rDNA V4 region of microeukaryotes, barcoded primers TAReuk454FWD1 5′-CCAGCASCYGCGGTAATTCC-3′ and TAReukREV3 5′-ACTTTCGTTCTTGATYRA-3′ (38) were used for amplification. The amplification procedure consisted of an initial denaturation step of 98°C for 30 s, followed by three-step amplification for 35 cycles consisting of denaturation at 98°C for 15 s, annealing at 56°C for 30 s, and extension at 72°C for 1 min, followed by a final extension at 72°C for 10 min. VAHTSTM DNA Clean Beads (Vazyme, Nanjing, China) were used to purify the PCR products. Finally, libraries were sequenced using the NovaSeq 6000 PE250 platform (Illumina, Novaseq 6000, USA).

### Sequence analyses

After sequencing, DADA2 (Divisive Amplicon Denoising Algorithm 2) v1.6.0 (39) of QIIME 2 (40) was used to filter low-quality reads, denoise sequences, merge read pairs, and remove chimeras, followed by precise amplicon sequence variant (ASV) classification and enumeration in the form of an ASV count table. The taxonomic assignments for microeukaryotes and prokaryotes were performed using QIIME 1 (41) with the Silva 138 database (42). After the generation of the ASV table, sequences higher than three were retained, and the representative sequences of metazoa and plants (Rhodophyta, Streptophyta, and Ulvophyceae) were removed from the microeukaryotic data set. The ASV tables for prokaryotes and microeukaryotes were rarefied to 24,743 and 4,013 reads per sample for subsequent analyses, respectively (Table S2). All raw sequences in this study have been deposited at the NCBI Sequence Read Archive under the accession number PRJNA1337497.

### Microbial diversity analysis

The alpha diversity indices, including the observed richness (number of ASVs), Shannon–Wiener index (43), and Pielou’s evenness index (44), were calculated for each sample to compare the differences in alpha diversity among various groups. The Kruskal–Wallis test, along with the Wilcoxon test for pairwise comparisons, was used to test the differences in diversity indices among various groups (45, 46). Principal coordinate analysis (PCoA) was performed to explore differences in microbial structures for groups based on community dissimilarity (Bray–Curtis distance) (47), and significant differences between groups were assessed using PERMANOVA (48). All above analyses were performed in R software (49) with the “vegan” package (50).

### Relationships between microbial communities and physicochemical factors

Measured environmental factors were log (*x* + 1) transformed to improve normality and homoscedasticity, except pH. Canonical correspondence analysis (CCA) was performed to explore the relationships between microbial community structures and physicochemical factors based on detrended correspondence analysis (DCA). The longest gradient lengths of DCA exceeded 4.0, indicating that the majority of species exhibited a unimodal response to physicochemical variation (51). A Monte Carlo permutation test was performed to explore the correlation coefficients and their corresponding significance between variations in microbial community structures and changes in physicochemical factors (52).

### Network analysis

The co-occurrence networks for microorganisms were conducted using the R package “Hmisc” (53) and “igraph” (54). For each network, the ASVs that appeared in more than half of the samples were retained for analysis, then were visualized and analyzed independently per group (EZ, MZ, BZ, and R1–R5). The R package “Hmisc” was used to calculate pairwise Spearman’s rank correlations (*r*) between two ASVs (53). Only robust (|*r*| > 0.6) and statistically significant (*P* < 0.01) correlations were incorporated in the network analyses. Network visualization was produced with Gephi version 0.9.2 (55). Simultaneously, 1,000 Erdös–Réyni random networks were generated for comparison with the topology of a real network, with each edge possessing an equal probability of being assigned to any node (56). The topological properties of real and random networks, including clustering coefficient, average path length, modularity, graph density, network diameter, and average degree (AD), were compared to determine the randomness of real networks using the R package “igraph” (52). Utilizing metabolic network approaches (57), the network hubs (*Zi* > 2.5, *Pi* > 0.62), module hubs (*Zi* > 2.5, *Pi* < 0.62), connectors (*Zi* < 2.5, *Pi* > 0.62), and peripherals (*Zi* < 2.5, *Pi* < 0.62) were identified (58). Network stability was evaluated by robustness, which was defined as the proportion of the remaining species in this network after random or targeted node removal (59). Spearman correlations were calculated to identify the relationship between topological parameters and environmental factors using the “picante” R package (60).

### Analysis of ecological processes

Null-model theory based on phylogenetic structure was applied to investigate the microbial community assembly processes. The statistical framework used in this study was proposed in a previous study (61) and calculated based on 999 randomized permutations using the “picante” package (60) in R software (49). First, we calculated the beta mean nearest taxon distance metric (βMNTD) by assessing the phylogenetic turnover between each pair of microbial communities (62). Second, we calculated the β-nearest taxon index (βNTI), which is the difference between the observed βMNTD and the mean of the null distribution of βMNTD normalized by its standard deviation. A significant deviation (i.e., |βNTI| > 2) indicates the dominance of deterministic processes: βNTI values > +2 indicate significantly more than expected phylogenetic turnover (variable selection), whereas βNTI values < –2 indicate significantly less than expected phylogenetic turnover (homogeneous selection).

If the observed βMNTD values do not significantly deviate from the null βMNTD distribution (|βNTI| < 2), this indicates that the observed variation in phylogenetic community composition is not the result of deterministic processes, but rather of stochastic processes (dispersal limitation and homogenizing dispersal) or an undominated process (moderate dispersal and weak selection) (62). To differentiate these processes, we computed the pairwise Raup–Crick dissimilarities (RC_bray_) between samples as described in a previous study (63). RC_bray_ values < −0.95 indicate microbial communities affected by homogenizing dispersal, whereas dispersal limitation results in RC_bray_ > 0.95. For RC_bray_ < |0.95|, moderate dispersal and weak selection result in an undominated process.

We explored the effect of deterministic processes and stochastic processes on patterns of microbial distribution and co-occurrence by examining the checkerboard score (C-score) (64). The values obtained were standardized to allow comparisons among assemblages using the standardized effect size (SES). The SES for the C-score was estimated as the difference between the observed index and the mean of the simulated index, divided by the standard deviation of the simulated index (65). The C-score was calculated based on 30,000 simulations and the sequential swap randomization algorithm with the “EcoSimR” package (66).

## RESULTS

### Environmental characteristics

Environmental factors exhibited significant variation along the vertical profile of the WC and throughout the different benthic habitats in the SEs (Fig. S1 and S2). Except for NO_2_^−^, the measured environmental factors exhibited significant variations among the different water layers, and all measured environmental factors revealed significant differences among the varying benthic habitats. The concentrations of DSi, NO_3_^−^, and PO_4_^3−^ showed a significantly increasing trend from epipelagic zone (EZ) to bathypelagic zone (BZ) of WC, whereas Sal for EZ was lower than that for mesopelagic zone (MZ) and BZ. By contrast, the concentrations of Temp showed a significant decreasing trend from EZ to BZ of WC, and the concentrations of DO and DOC for EZ were significantly higher than those for MZ and BZ (Fig. S1). In addition, the majority of measured environmental factors for the sediments from the shellfish habitats (R1 and R5) appeared to be higher than those of other habitats (R2, R3, and R4), except for DSi (Fig. S2).

### Diversity and distribution patterns of microbial communities

**Fig 2:**
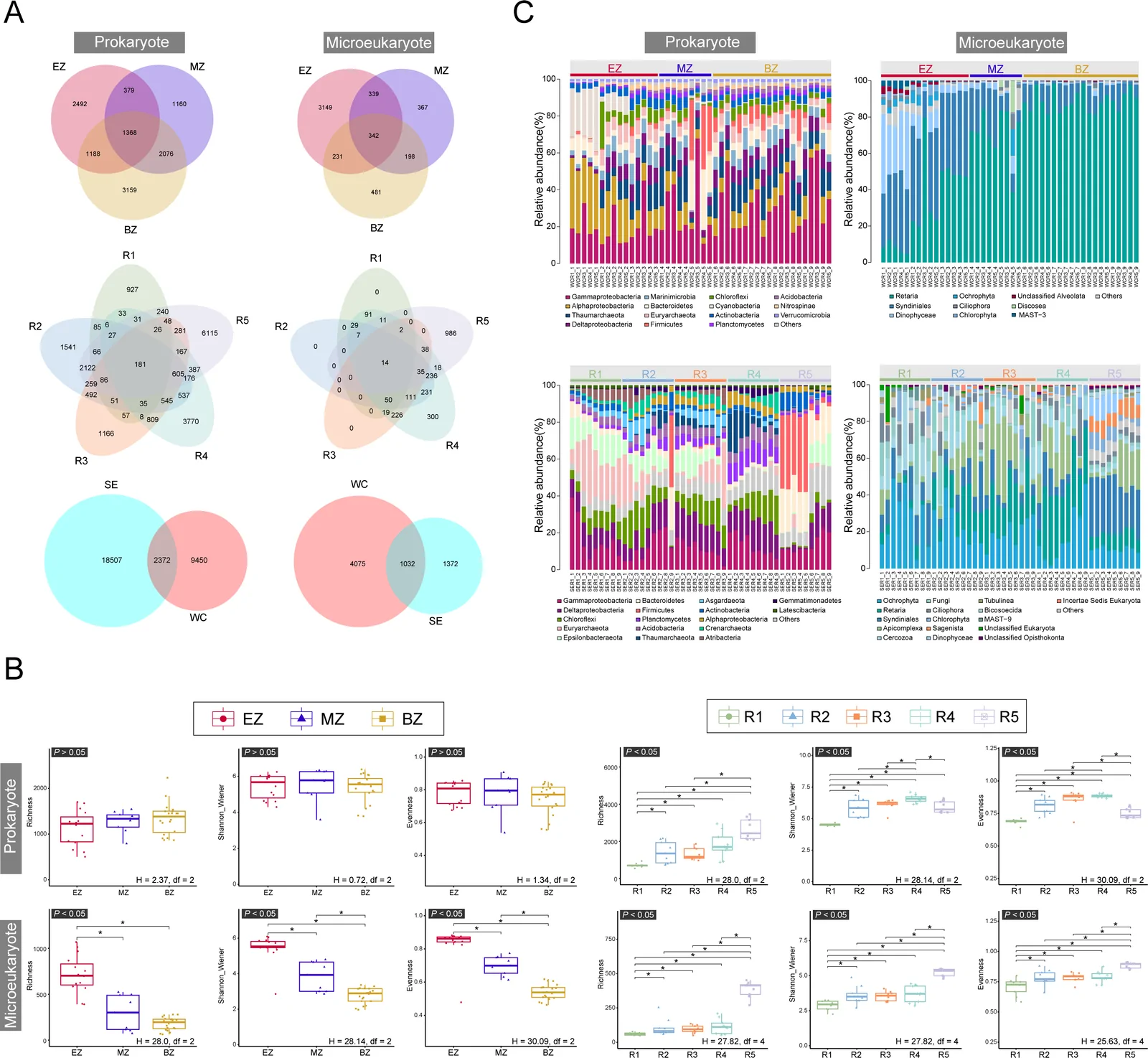
(**A**) Venn diagrams illustrating the shared and unique ASVs across three water layers and five benthic habitats, as well as between the water column and sediments, between prokaryotic and microeukaryotic communities. (**B**) The boxplots for richness, Shannon–Wiener, and Pielou’s evenness indices of prokaryotic and microeukaryotic communities across three water layers and five benthic habitats. “*” Represents a significant difference (*P* < 0.05) between groups. (**C**) Taxonomic profile of prokaryotic and microeukaryotic ASVs of every sample type across three water layers and five sediment habitats. EZ, epipelagic zone; MZ, mesopelagic zone; BZ, bathypelagic zone.

A total of 30,329 prokaryotic ASVs (PASVs) and 6,479 microeukaryotic ASVs (EASVs) were detected. Among them, 1,368 PASVs (4.51%) and 342 EASVs (5.28%) were shared among samples across three water layers (EZ, MZ, and BZ; Fig. 2A). Besides, 181 PASVs (8.88%) and 14 EASVs (2.86%) were shared among samples across five habitats (R1, R2, R3, R4, and R5), and 2,372 PASVs and 1,032 EASVs were shared between samples from WC and SE, respectively (Fig. 2A). The richness, Shannon–Wiener, and Pielou’s evenness indices of microeukaryotic communities showed significant differences across three water layers and five benthic habitats (*P* < 0.05). By contrast, the prokaryotic indices demonstrated significant differences across the five benthic habitats but no significant differences across the three water layers (Fig. 2B). In addition, the indices of alpha diversity of microeukaryotes showed a significant decreasing trend from EZ to BZ. Furthermore, most of the alpha diversity indices for prokaryotes and microeukaryotes were highest in R5 compared with other benthic habitats (Fig. 2B).

Histograms showing the most frequently detected prokaryotic and microeukaryotic taxa (with relative abundance higher than 0.5%) across samples from three water layers (EZ, MZ, and BZ) and five sediment habitats (Fig. 2C). For prokaryotes, Gammaproteobacteria comprised the most abundant group in the water column, with an average relative abundance of 26.80%, followed by Alphaproteobacteria (12.67%), Thaumarchaeota (10.17%), Deltaproteobacteria (7.66%), Marinimicrobia (6.40%), and Bacteroidetes (6.06%). Among the dominant taxa, Gammaproteobacteria, Thaumarchaeota, and Deltaproteobacteria exhibited relatively higher relative abundances in EZ, Firmicutes were relatively abundant in MZ, whereas Alphaproteobacteria and Cyanobacteria were relatively enriched in BZ. Across the five sediment habitats, the predominant ASVs were assigned to Gammaproteobacteria (13.52%), Deltaproteobacteria (11.73%), Chloroflexi (9.34%), Euryarchaeota (8.55%), and Epsilonbacteraeota (7.28%). This type of habitat also exerted a pronounced influence on the composition of prokaryotic communities. For instance, Gammaproteobacteria and Euryarchaeota showed relatively high abundances in the mussel habitat, Planctomycetota and Thaumarchaeota were relatively enriched in the dead clam habitat, and Bacteroidota and Firmicutes were relatively dominant in the clam habitat. For microeukaryotes, Retaria (65.51%), Syndiniales (21.77%), and Dinoflagellata (6.27%) were the three dominant groups, collectively accounting for nearly 93.55% of the total microeukaryotic sequences. EZ harbored a more diverse microeukaryotic community than BZ, characterized by elevated relative abundances of Syndiniales and Dinoflagellata in EZ, whereas Retaria was predominant in BZ. A substantial difference in microeukaryotic community structure was also observed among the five benthic habitats, with Ochrophyta, Retaria, Syndiniales, Apicomplexa, and Cercozoa exhibiting elevated relative abundances at the mussel habitat (R1), non-fauna seabed habitat (R3), dead clam habitat (R4), and clam habitat (R5), respectively.

**Fig 3:**
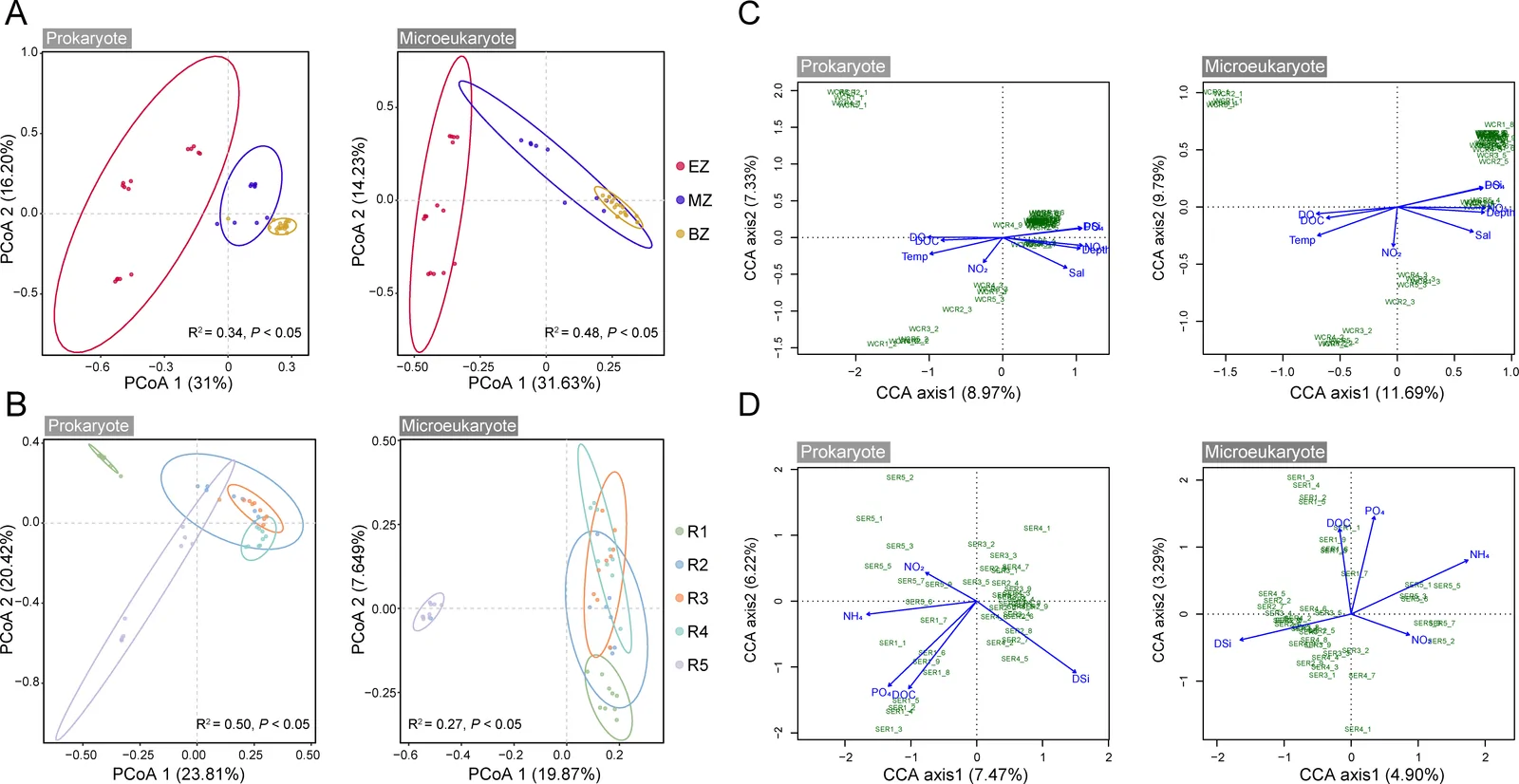
Principal coordinate analysis (PCoA) of the prokaryotic and microeukaryotic communities across three water layers (**A**) and five benthic habitats (**B**). Canonical correspondence analysis (CCA) for prokaryotic and microeukaryotic community compositions in the water columns (**C**) and sediments (**D**). EZ, epipelagic zone; MZ, mesopelagic zone; BZ, bathypelagic zone; Temp, temperature; Sal, salinity; DO, dissolved oxygen; DOC, dissolved organic carbon; DSi, dissolved silicate; NO_2_^−^, nitrite nitrogen; NO_3_^−^, nitrate nitrogen; PO_4_^3−^, phosphate phosphorus.

PCoA was used to evaluate the differences in the prokaryotic and microeukaryotic communities among different water layers and sediment habitats (Fig. 3A and 3B). In the water column, the PCoA results explained 47.20% and 45.86% of the variation in prokaryotic and microeukaryotic community composition, respectively. Meanwhile, PCoA revealed three distinct clusters of prokaryotic (*R*^2^ = 0.34, *P* < 0.05) and microeukaryotic communities (*R*^2^ = 0.48, *P* < 0.05), corresponding to EZ, MZ, and BZ. Notably, within the EZ, the samples further subdivided into three distinct sub-clusters corresponding precisely to the specific sampling depths (5 m, 100 m, and 200 m), highlighting pronounced vertical heterogeneity within the euphotic zone (Fig. 3A). In the sediment, the first two PCoA axes accounted for 44.23% and 27.51% of the total variation in prokaryotic (*R*^2^ = 0.50, *P* < 0.05) and microeukaryotic (*R*^2^ = 0.34, *P* < 0.05) community compositions, respectively. The PCoA results clearly demonstrated distinct community compositions among sediment habitats, with samples from the same benthic habitat clustering together.

### Relationships between microbial community structures and environmental factors

CCA was performed to explore the relationship between environmental factors and prokaryotic and microeukaryotic community structures. In the water columns, the first two axes collectively explained 16.30% and 21.48% of the overall variation in prokaryotic and microeukaryotic community structure, respectively (Fig. 3C). In the sediment, the first two axes collectively explained 13.69% and 8.19% of the overall variation in prokaryotic and microeukaryotic community structure, respectively (Fig. 3D). Mantel test results showed that several environmental factors were significantly correlated with microbial community structures. Among them, the measured environmental factors had significant correlations with microeukaryotic and prokaryotic community structures of water columns and sediments, except for NO_2_^−^ (Table 1). Furthermore, environmental factors had stronger correlations with the structures of microeukaryotic communities compared with prokaryotic communities in water columns, whereas the opposite pattern was found in sediment (Table 1). For instance, the average correlation coefficient between microeukaryotic community structures (*r* = 0.826) and measured environmental factors was higher than that of prokaryotic community structures (*r* = 0.633) in water columns, and the average correlation coefficient between measured environmental factors and prokaryotic community structures (*r* = 0.496) was higher than that of microeukaryotic counterparts (*r* = 0.409) in sediments (Table 1).

**TABLE 1:** Monte Carlo permutation test between prokaryotic or microeukaryotic communities and environmental factors^*a*^

Sample type and parameter	Prokaryotes		Microeukaryotes	
	*r*	*P*	*r*	*P*
WC				
Depth	0.872	0.001*	0.971	0.001*
Temp	0.720	0.001*	0.936	0.001*
Sal	0.848	0.001*	0.830	0.001*
DO	0.608	0.001*	0.836	0.001*
DOC	0.374	0.001*	0.650	0.001*
DSi	0.701	0.001*	0.989	0.001*
NO_2_^−^	0.071	0.193	0.247	0.007*
NO_3_^−^	0.808	0.001*	0.986	0.001*
PO_4_^3+^	0.695	0.001*	0.993	0.001*
SE				
DOC	0.562	0.001*	0.380	0.001*
DSi	0.579	0.001*	0.433	0.001*
NO_2_^−^	0.133	0.060	0.129	0.103
NH_4_^+^	0.518	0.001*	0.594	0.001*
PO_4_^3+^	0.688	0.001*	0.508	0.001*

^*a*^ Significances are tested based on 999 permutations. WC, water column sample; SE, sediment samples; Temp, temperature; Sal, salinity; DO, dissolved oxygen; DOC, dissolved organic carbon; DSi, dissolved silicate; NO_2_^−^, nitrite nitrogen; NO_3_^−^, nitrate nitrogen; PO_4_^3−^, phosphate phosphorus. *P< 0.05 is considered a significant effect and labeled in bold.

### Microbial co-occurrence networks in water columns and sediments

**Fig 4:**
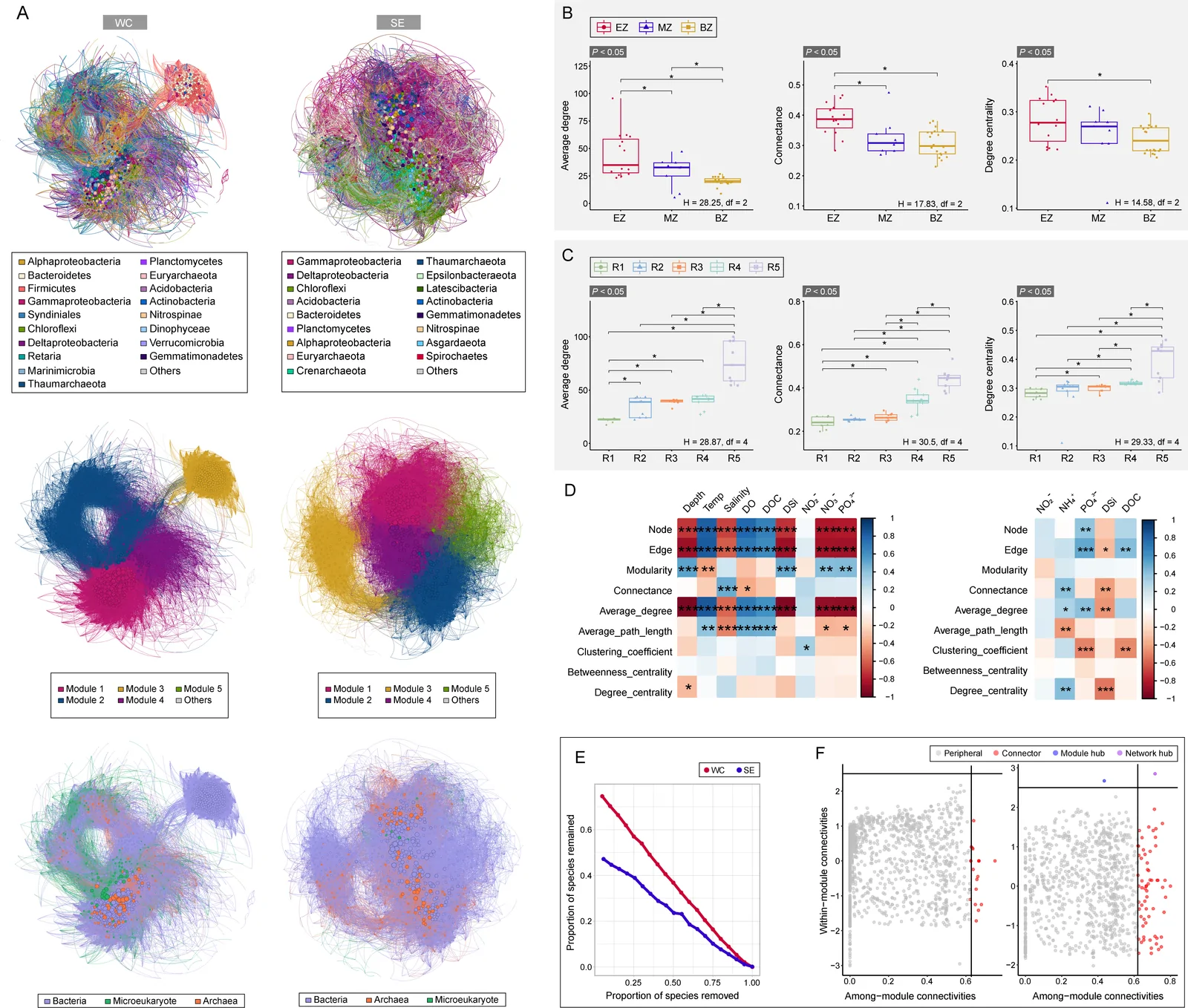
Co-occurrence patterns of prokaryotic and microeukaryotic communities based on node-level network analysis. Nodes are colored according to different taxonomic groups, modules, and domains (**A**). Comparison of node-level topological features among co-occurrence networks from the three water layers (**B**) and five sediment habitats (**C**). “*” Represents a significant difference between groups (*P* < 0.05). Spearman correlations between the topological features and environmental factors (**D**). Significance levels are denoted with *P < 0.05, **P < 0.01, and ***P < 0.001. Network robustness of microbial communities from water columns and sediments (**E**). The nodes of co-occurrence networks were separated by their within-module connectivity (*Zi*) and among-module connectivity (*Pi*) (**F**). WC, water column samples; SE, sediment samples; EZ, epipelagic zone; MZ, mesopelagic zone; BZ, bathypelagic zone.

The microbial networks from water columns and sediments mostly consisted of positive correlations (92.01% and 98.55%) than negative ones (7.99% and 1.45%; Fig. 4A and Table 2). The microbial network from water columns was more complex than that in sediments. The microbial network from water columns was composed of 1,241 nodes with 80,295 edges, whereas that of sediments contained 938 nodes linked by 40,204 edges (Table 2). Furthermore, the prokaryotic and microeukaryotic networks from water columns were more complex than those of sediments in terms of elevated values of edges and AD (Table S3). The microbial network from water columns primarily consisted of Alphaproteobacteria (11.67%), Bacteroidetes (9.33%), Firmicutes (9.17%), Gammaproteobacteria (8.85%), Syndiniales (7.80%), Chloroflexi (6.60%), Deltaproteobacteria (6.19%), Retaria (5.55%), Marinimicrobia (5.31%), Thaumarchaeota (5.15%), and Planctomycetes (5.15%). The predominant taxonomic groups of prokaryotic and microeukaryotic networks in water columns were similar to those of the microbial network in water columns (Fig. S3A and S4A). The microbial network from sediments predominantly comprised Gammaproteobacteria (15.3%), Deltaproteobacteria (13.92%), Chloroflexi (12.22%), Acidobacteria (6.38%), and Bacteroidetes (6.27%; Fig. 4A). The dominant taxonomic groups of the prokaryotic network from sediments were similar to those of the microbial network from sediments, whereas the dominant taxonomic groups of the microeukaryotic network from sediments exhibited greater diversity than the microbial network from sediments (Fig. S3A and S4A). Five dominant modules (relative abundance >10%) were identified in the networks from water columns and sediments (Fig. 4A). The microbial network from water columns was composed of bacteria (75.30%), microeukaryotes (16.17%), and archaea (8.53%), and the sediment network was composed of bacteria (84.17%), archaea (12.65%), and microeukaryotes (3.19%; Fig. 4A). The intra-domain correlations dominated the species interactions in networks from the water columns and sediments (Table S4). Bacterial correlations were the most abundant species interactions within networks in water columns (64.04%) and sediments (69.45%). Aside from bacterial correlations, the dominant correlations (relative abundance >10%) of networks in water columns included archaea–bacteria (16.56%) and bacteria–microeukaryote (12.72%), but only archaea–bacteria correlations (25.64%) were present in sediments (Table S4). Furthermore, the predominant correlations across phyla within the same domain varied between water columns and sediments. The most abundant correlations between bacterial phyla were Bacteroidetes–Firmicutes (8.69%), whereas that in sediments was Deltaproteobacteria–Gammaproteobacteria (4.35%; Table S4).

**TABLE 2:** Comparison of topological properties between prokaryotic and microeukaryotic co-occurrence networks^a^

Network	*N*	*E*	CC	APL	MD	GD	ND	AD
WC	1,241	80,295	0.735	3.628	0.578	0.104	10	129.404
SE	938	40,204	0.562	2.840	0.462	0.091	7	85.723

^a^ *N*, number of nodes; *E*, number of edges; CC, clustering coefficient; APL, average shortest path length; MD, modularity; GD, graph density; ND, network diameter; and AD, average degree.

Moreover, the average degree, connectance, and degree centrality of the network from EZ were significantly higher than those of BZ (Fig. 4B). The average degree, connectance, and degree centrality of the network from R5 were significantly (*P* < 0.05) higher than those of other benthic habitats, and those of R1 were significantly (*P* < 0.05) lower than those of other benthic habitats (Fig. 4C). This pattern was consistently observed in networks of prokaryotes and microeukaryotes from water columns and sediments (Fig. S3C and S4C). The measured environmental factors showed significant correlations (*P* < 0.05) with several topological parameters of the network from water columns and sediments, except for NO_2_^−^ (Fig. 4D). This pattern was consistently observed in networks of prokaryotes and microeukaryotes from water columns and sediments (Fig. S3B and S4B). Robustness analysis demonstrated that the microbial network from water columns had higher robustness than that from sediments, indicating that the stability of the microbial network in water columns was higher than that in sediments (Fig. 4E). In total, 19 PASVs and 11 EASVs were identified as connectors to maintain the microbial network from water columns (Fig. 4F and Table S4). Among them, the majority of PASVs were assigned to Alphaproteobacteria (AEGEAN-169 and SAR11 clade I), Deltaproteobacteria (OM27 clade, P3OB-42 and NB1-j), and Gammaproteobacteria (*Woeseia*, EPR3968-O8a-Bc78, UBA10353, and Milano-WF1B-44). The majority of EASVs were assigned to Syndiniales (*Duboscquella* and Syndiniales Group I) and Retaria (*Cladococcus*, Acantharea Group II, and Unclassified Chaunocanthida; Table S5). A total of 75 PASVs and 5 EASVs were identified as keystone species to maintain the microbial network from sediments (Fig. 4F and Table S4). The network hub was assigned to Deltaproteobacteria (*Desulfobulbus*), the module hub was assigned to Bacteroidetes (*Cytophaga*), and the remaining keystone species were connectors. Among them, the majority of PASVs were assigned to Gammaproteobacteria (*Woeseia*, *Nitrosomonas*, *Colwellia*, *Cupriavidus*, *Psychromonas*, *Thiogranum*, *Thiohalophilus*, B2M28, Unclassified Ectothiorhodospiraceae, Unclassified Gammaproteobacteria, JL-ETNP-Z34, Methylomonaceae Group 2, pItb-vmat-80, Unclassified Thiohalorhabdaceae, Unclassified Thiomicrospiraceae, and Unclassified Thiotrichaceae), Deltaproteobacteria (*Desulfatiglans*, *Desulfocapsa*, *Desulfofrigus*, *Desulfuromonas*, *Geopsychrobacter*, Unclassified Desulfobacteraceae, Unclassified Desulfobulbaceae, MBNT15, NB1-j, and Unclassified Sandaracinaceae), and Epsilonbacteraeota (*Sulfurimonas* and *Sulfurovum*; Table S5). The EASVs for connectors were assigned to several taxonomic groups, including Ciliophora (*Maryna*), MAST-9, Ochrophyta (*Hydrurus*), Retaria (RAD B), and Syndiniales (Syndiniales Group I; Table S5).

### Microbial co-occurrence networks across water layers and benthic habitats

**Fig 5:**
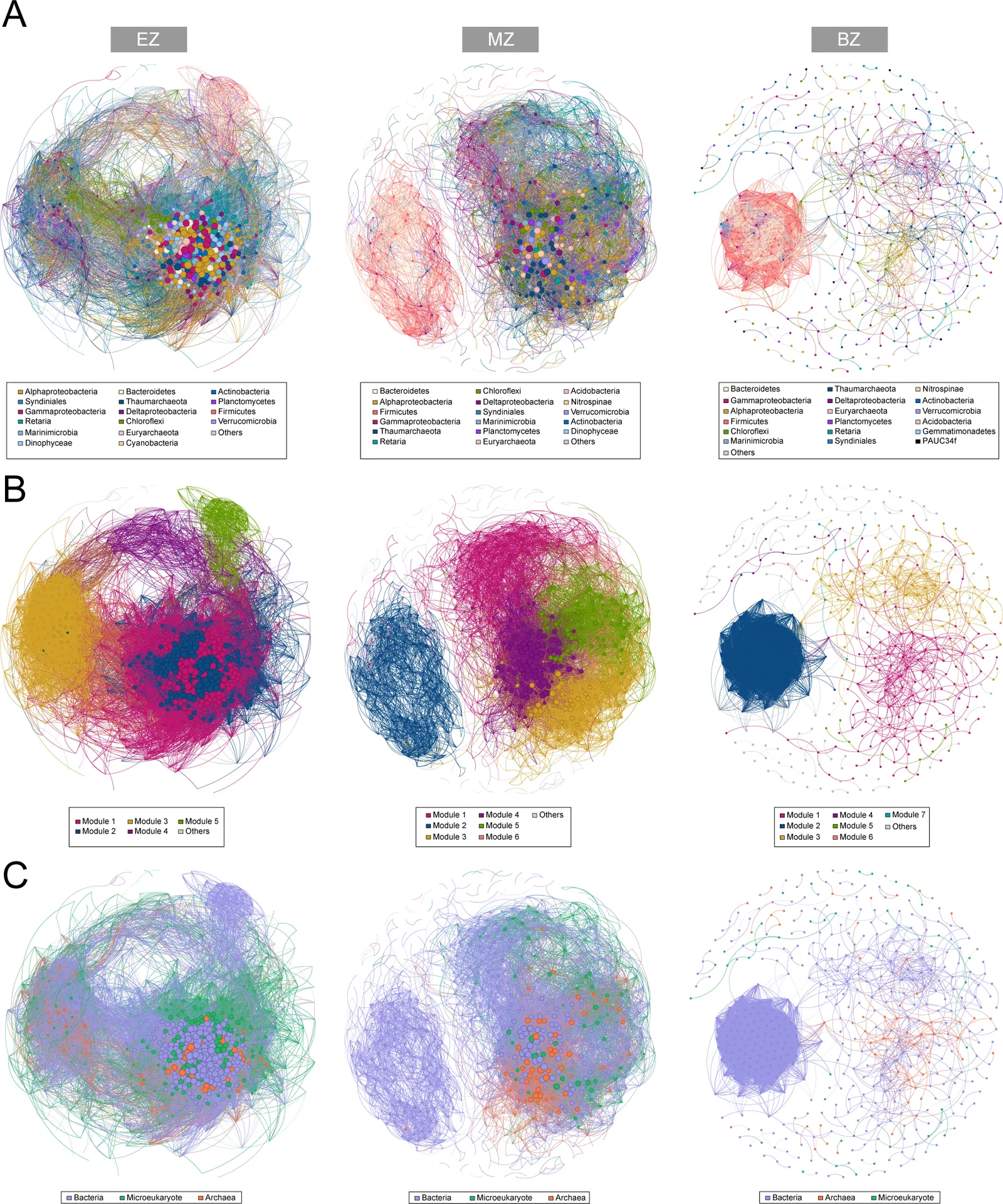
Co-occurrence patterns of microbial communities across the three water layers based on network analysis. Nodes are colored according to different taxonomic groups (**A**), modules (**B**), and domains (**C**). EZ, epipelagic zone; MZ, mesopelagic zone; BZ, bathypelagic zone.

The microbial network of EZ from water columns was more complex than that of MZ and BZ in terms of elevated values of edges and AD (Fig. 5 and Table 3). Alphaproteobacteria was the most abundant taxonomic group in microbial networks of EZ (18.53%), whereas Bacteroidetes was the most abundant taxonomic group in microbial networks of MZ (11.02%) and BZ (10.99%; Fig. 5A). There were five, six, and seven dominant modules (relative abundance >10%) in networks from EZ, MZ, and BZ, respectively (Fig. 5B). The microbial network of EZ was composed of bacteria (58.62%), microeukaryotes (33.48%), and archaea (7.90%), whereas the networks of MZ and BZ were composed of bacteria (74.28% and 81.61%), microeukaryotes (14.70% and 10.57%), and archaea (11.02% and 7.82%), respectively (Fig. 5C). The bacterial correlations increased (from 44.37% to 95.80%) with water depth, whereas the bacteria–microeukaryote correlations showed the opposite pattern in water columns (from 32.69% to 0.40%; Table S6). Furthermore, the most abundant phylum correlation for the EZ network was Alphaproteobacteria–Alphaproteobacteria (6.20%), whereas that of MZ and BZ was Bacteroidetes–Firmicutes (4.97% and 31.27%, respectively; Table S6). A total of 1, 135, and 15 keystone species were found in EZ, MZ, and BZ, respectively (Table S7). Among them, 1 connector assigned to Retaria was found in EZ, 1 module hub and 134 connectors were found in MZ, and 3 module hubs and 12 connectors were found in BZ (Table S7). Bacteroidetes (26 PASVs), Retaria (6 EASVs), and Syndiniales (6 EASVs) dominated the prokaryotic and microeukaryotic keystone species of MZ. Gammaproteobacteria (four PASVs), Retaria (one EASV), and Syndiniales (one EASV) dominated the prokaryotic and microeukaryotic keystone species of BZ (Table S7).

**TABLE 3:** Topological properties of networks of WC and SE across different vertical and horizontal gradients^a^

Category	Group	*N*	*E*	CC	APL	MD	GD	AD
WC	EZ	689	23,586	0.710	4.076	0.623	0.010	68.464
	MZ	735	10,341	0.531	3.301	0.483	0.038	28.139
	BZ	437	5,438	0.898	4.870	0.198	0.057	24.888
SE	R1	178	155	0.771	1.429	0.965	0.010	1.742
	R2	666	3,094	0.614	6.567	0.754	0.014	9.291
	R3	400	557	0.676	3.050	0.930	0.007	2.785
	R4	991	5,898	0.621	5.108	0.754	0.012	11.903
	R5	2,095	167,459	0.671	2.631	0.351	0.076	159.865

^a^ *N*, number of nodes; *E*, number of edges; CC, clustering coefficient; APL, average shortest path length; MD, modularity; GD, graph density; and AD, average degree.

**Fig 6:**
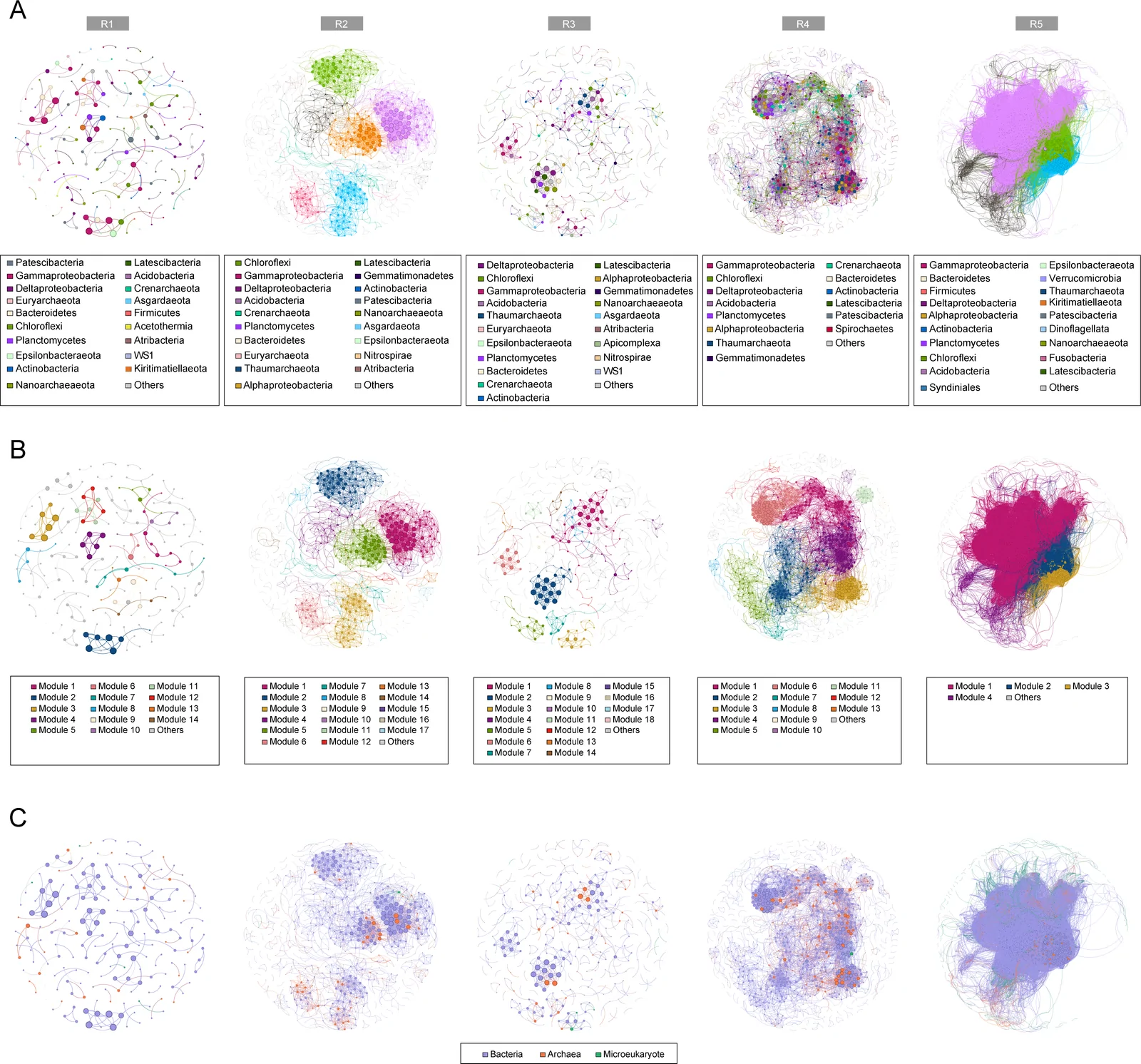
Co-occurrence patterns of microbial communities across the five benthic habitats based on network analysis. Nodes are colored according to different taxonomic groups (**A**), modules (**B**), and domains (**C**).

The microbial network of R5 from sediments was the most complex, whereas that of R1 was the simplest compared with other benthic habitats in terms of values of edges and AD (Fig. 6 and Table 3). The dominant taxonomic groups (relative abundance >10%) of R1 and R5 were different from those of R2, R3, and R4 (Fig. 6A). Patescibacteria (11.68%), Gammaproteobacteria (11.17%), and Deltaproteobacteria (10.66%) dominated the network of R1, whereas Gammaproteobacteria (16.6%), Bacteroidetes (16.41%), and Firmicutes (12.31%) dominated the network of R5. However, Deltaproteobacteria (mean of 12.93), Chloroflexi (mean of 12.87%), and Gammaproteobacteria (mean of 12.11%) dominated the networks of R2 to R4 (Fig. 6A). In networks R1–R5, there were 14, 17, 18, 13, and 4 dominant modules (relative abundance >10%), respectively (Fig. 6B). The microbial networks of R1–R4 were composed of bacteria (mean of 83.60%), archaea (14.43%), and microeukaryotes (1.91%), whereas the network of R5 was composed of bacteria (86.94%), microeukaryotes (9.05%), and archaea (4.01%; Fig. 6C). The bacterial correlations dominated species interactions in networks R1–R5 (Table S6). The most abundant phylum correlation for the R1 and R5 networks was Bacteroidetes–Gammaproteobacteria (10% and 9.8%, respectively), whereas that for R2–R4 was within the Proteobacteria (mean of 3.71%; Table S6). A total of 6, 14, 2, 33, and 982 keystone species were found from R1 to R5, respectively (Table S7). Among them, 6 module hubs were found in R1, 14 connectors were found in R2, 2 connectors were found in R3, 1 module hub and 32 connectors were found in R4, and 7 network hubs and 975 connectors were found in R5 (Table S7). Microeukaryotic keystone species were exclusively found in R5. Gammaproteobacteria (3 PASVs) dominated the keystone species in R1, Acidobacteria (5 PASVs) dominated the keystone species in R2, 2 connectors were assigned to Chloroflexi and Gemmatimonadetes in R3, Deltaproteobacteria (6 PASVs) dominated the keystone species in R4, and Firmicutes (198 PASVs) and Syndiniales (17 EASVs) dominated the prokaryotic and microeukaryotic keystone species in R5 (Table S7).

### Ecological processes driving the prokaryotic and microeukaryotic communities

**Fig 7:**
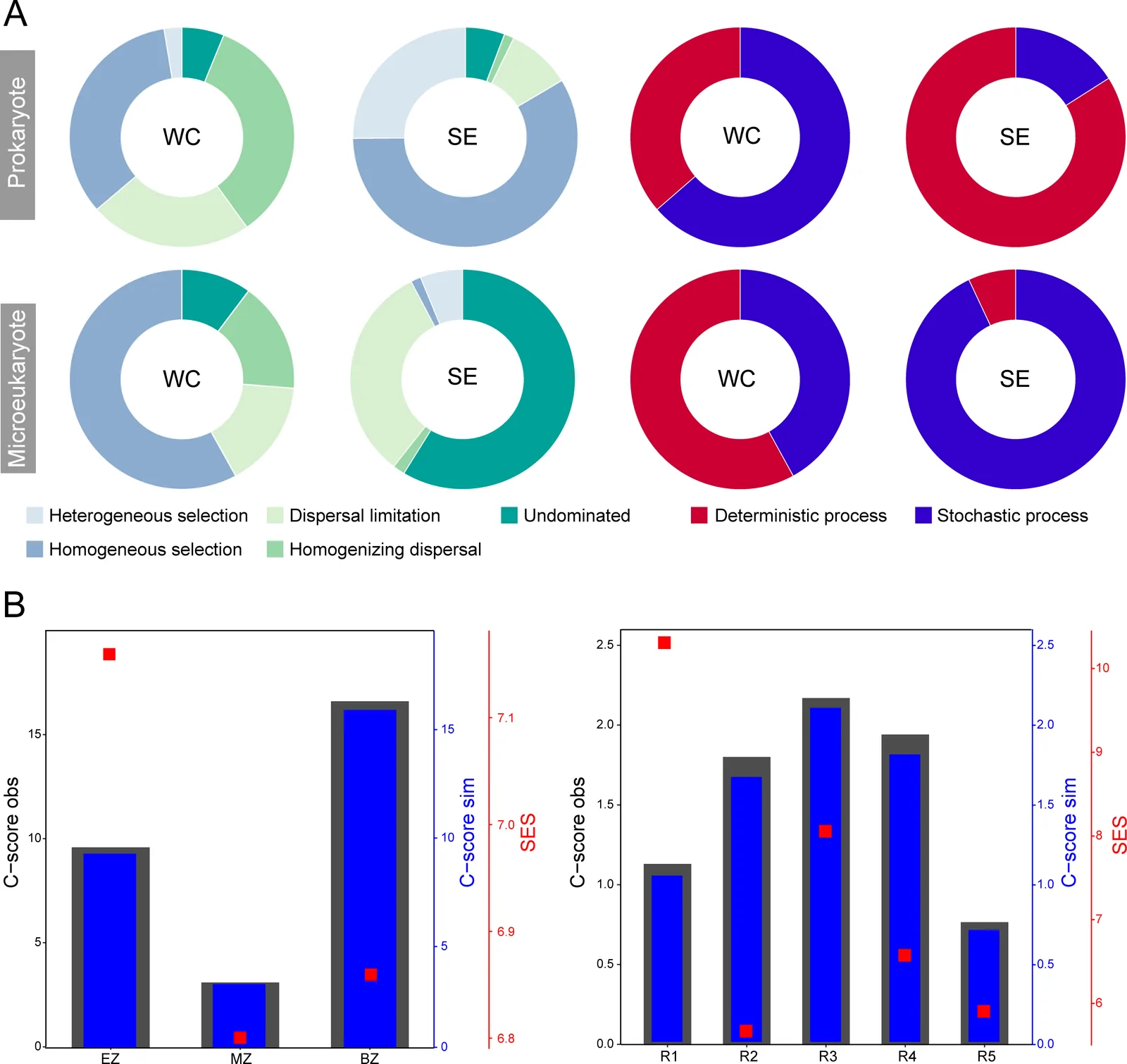
(**A**) Relative importance of ecological processes to the community assembly of prokaryotic and microeukaryotic communities from the water columns and sediments. WC, water column samples; SE, sediment samples. (**B**) Results of C-score and SES of microbial networks from water columns and sediments.

The null model demonstrated that the deterministic and stochastic processes significantly influenced the architecture of the prokaryotic and microeukaryotic communities (Fig. 7A). The deterministic processes played a more important role in structuring the microeukaryotic communities in water columns (58%) and prokaryotic communities in sediments (84%) than the stochastic processes, whereas the stochastic processes played an important role in structuring prokaryotic communities in water columns (63.62%) and microeukaryotic communities in sediments (93%) than the deterministic processes (Fig. 7A). The prokaryotic communities in water columns were predominantly influenced (relative importance >10%) by homogeneous dispersal (34.15%), homogeneous selection (33.83%), and dispersal limitation (23.40%). The microeukaryotic communities in water columns were primarily governed by homogeneous selection (58%), homogeneous dispersal (16%), dispersal limitation (16%), and undominated factors (10%; Fig. 7A). The prokaryotic communities in sediments were dominated by homogeneous selection (59%) and heterogeneous selection (25%), whereas the microeukaryotic communities in sediments were shaped by undominated (59%) and dispersal limitation (32%).

The SES values of microbial networks from water columns and sediments were higher than two, suggesting that their co-occurrence patterns were primarily influenced by deterministic processes rather than stochastic processes (Fig. 7B). The SES value of the microbial network for EZ was higher than that for MZ and BZ, indicating stronger deterministic impact on the microbial co-occurrence pattern for EZ compared with MZ and BZ (Fig. 4B). Moreover, the SES value of the microbial network for R1 was higher than that of other benthic habitats, indicating stronger deterministic impact on the microbial co-occurrence pattern for R1 compared with benthic habitats (Fig. 4B).

## DISCUSSION

### Distribution patterns of microbial communities in water columns and sediments

Our study first characterized the distribution patterns of microeukaryotic communities within water columns in the Haima cold seep and found that these communities showed vertical and horizontal distribution patterns in water columns and sediments, respectively (Fig. 2 and 3). The relative abundance of Syndiniales and Dinophyceae decreased but that of Retaria increased with rising depth in water columns (Fig. 2C). The dominance of Syndiniales and Dinophyceae may be attributed to these dinoflagellates’ various strategies for adjusting to environmental variations between EZ and MZ, along with their ability to switch between autotrophic and heterotrophic trophic modes (34). The prevalence of Retaria in deeper layers may be partially attributed to their siliceous skeleton, which can withstand the elevated pressures at these depths (67). The alpha diversities of microeukaryotes decreased with depth (Fig. 2B), indicating that environmental factors such as light, temperature, and dissolved oxygen play key roles in shaping microeukaryotic diversity. For example, light facilitates the proliferation of photosynthetic algae, and reduced levels of dissolved oxygen and temperature in deep water layers limit the metabolic rates of many microeukaryotes (36). The microeukaryotic communities from MZ and BZ were more similar than those from MZ and EZ, indicating a more fluid interchange of microeukaryotic individuals between MZ and BZ compared with MZ and EZ (Fig. 3). Furthermore, the distinct sub-clustering observed within the EZ underscores profound intra-zone heterogeneity (Fig. 3A). These sharp vertical transitions in light, temperature, and nutrient availability drive rapid microbial succession within the EZ, which was consistent with conclusions of previous studies (35, 36). The alpha and beta diversities of microeukaryotes were significantly different among the five benthic habitats (Fig. 2B and 3). The alpha and beta diversities of microeukaryotes from benthic habitats with mussels and clams (R1 and R5, respectively) were markedly different from those of other benthic habitats (R2, R3, and R4). According to the monoclimax theory, the faunal communities in cold seeps developed sequentially, with the clam (R5) serving as the first faunal colonizer for inhabitation (68–70). Consequently, the microbial communities from the initial habitat of faunal succession were diverse and distinctly different from those of subsequent succession processes, which were influenced by ecological processes such as environmental selection and dispersal (70). The benthic habitats of mussels always appeared in strong seepage zones with high concentrations of methane (34, 71), which may exert a substantial influence on environmental selection for microbial communities, resulting in reduced alpha diversities and distinct community structures compared with other benthic habitats.

Prokaryotic communities exhibited vertical and horizontal distribution patterns in water columns and sediments, respectively. However, their distribution patterns differed from those of microeukaryotes (Fig. 2 and 3). In our study, the relative abundance of Cyanobacteria decreased, whereas that of Proteobacteria increased with rising depth in water columns, which was similar to a previous study (7). However, the alpha diversities of prokaryotes were not significantly different among different water layers (Fig. 2B), which differed from a previous study (7). One possible reason for this discrepancy was that samples were collected from different sites, where distinct ecological processes, influenced by varying biogeochemical conditions, determined the community structures. The alpha diversities of prokaryotes exhibited no significant differences, whereas that of microeukaryotes decreased with depth (Fig. 2B). The evenness value for prokaryotes at R5 was the second lowest compared with other benthic habitats, whereas that of microeukaryotes was the highest (Fig. 2B). The most differentiated community structures of prokaryotes in benthic habitats were R1, whereas that of microeukaryotes was R5 (Fig. 3B). The differences in alpha and beta diversity between prokaryotes and microeukaryotes implied that the ecological processes driving their assembly varied between the water column and sediments.

### Co-occurrence patterns of microbial communities in water columns and sediments

The network results indicated different species correlations of microorganisms from water columns and sediments (Fig. 4 and Tables S3 and S5). The correlations among bacteria dominated (with relative abundance of correlations >10%) over species correlations, followed by archaea–bacteria correlations from the water columns and sediments (Fig. 4 and Table S4). The predominance of intra-domain correlations for prokaryotes could be attributed to their environmental preferences and niche specialization. A previous study indicated that obligate metabolic cross-feeding is common in nature, where many archaeal and bacterial species lack complete pathways for synthesizing essential metabolites, leading to interdependent relationships (72). The syntrophic correlations between ANME and SRB are crucial for methane metabolism in cold seeps. The low homologous recombination rates observed in ANME and SRB may limit genetic exchange across diverse taxonomic groups, thereby reinforcing the evolutionary and ecological boundaries between domains and promoting a network structure characterized by correlations within prokaryotes (31). Nonetheless, the bacteria–microeukaryote correlations were more abundant (12.72%) in water columns than in sediments (1.67%; Table S3 and Table S5), which could be attributed to different energy sources between the water column and sediment. The sediment is driven primarily by chemosynthetic energy, fostering the intra-domain correlations of prokaryotes with similar environmental preferences. The water column, influenced by seep fluids, is also supplemented by allochthonous organic matter from sinking particles. This diverse carbon source supports a wide range of heterotrophic bacteria, which subsequently attract microeukaryotic species and facilitate increased bacteria–microeukaryote interactions (73).

Furthermore, the keystone species differed between the water column and sediment networks. These differences implied that unique microbial groups were crucial to the biogeochemical cycles of each habitat (Table S5). The prokaryotic keystone species in the network from water columns were predominantly represented by Alphaproteobacteria (AEGEAN-169, SAR11 clade I), Deltaproteobacteria (NB1-j), and Gammaproteobacteria (*Woeseia*; Fig. 4A and Table S5). The predominant proteobacteria suggested that they have the potential to integrate carbon, nitrogen, and sulfur cycles in cold-seep water columns (74). Retaria and Syndiniales predominated the microeukaryotic keystone species of the network from water columns (Fig. 4A). Retaria was one of the major players exporting organic carbon to the deep sea (75, 76), and they also act as hosts for many living microorganisms, such as Syndiniales (77). This parasitic relationship between Retaria and Syndiniales in water columns may be pivotal in maintaining the microbial ecosystems of deep-sea cold seeps (78). The sediment network hub associated with *Desulfobulbus* (Deltaproteobacteria) indicated that sulfate-reducing activity is crucial in maintaining the microbial network in sediments (79). Furthermore, keystone species, including *Nitrosomonas* and *Thiohalophilus* (Gammaproteobacteria), Desulfobacteraceae, Desulfobulbaceae, *Desulfatiglans* (Deltaproteobacteria), *Sulfurimonas*, and *Sulfurovum* (Epsilonbacteraeota), indicated that diverse metabolic potentials play important roles in maintaining microbial networks within sediments, including sulfate reduction, sulfur oxidation, denitrification, and ammonia oxidation (80–82). In contrast to the pronounced parasitic relationships observed in microbial networks from water columns, the microeukaryotic keystone species within sediment networks exhibit greater diversity of trophic modes, including parasitic (Syndiniales), autotrophic (Ochrophyta), heterotrophic (Ciliophora and MAST-9), and mixotrophic (Retaria) lifestyles. Thus, microeukaryotes in sediments may adapt to the deep-sea cold seep environment via diverse species interactions more than their water counterparts (Fig. 4 and Table S5).

The microbial networks also showed vertical and horizontal patterns across water columns and sediments (Fig. 4–6). The complexity of networks was highest in EZ, with the microbial network from the clam bed (R5) exhibiting the highest complexity, while that from the mussel bed (R1) displayed the lowest complexity compared with the other habitats (R2–R4; Fig. 6). The highest network complexity in EZ could be attributed to the vertical gradient in energy availability. In EZ of oceans, photosynthetic primary production by phytoplankton and cyanobacteria provides a rich, diverse foundation of organic carbon, supporting a wide array of heterotrophic bacteria and microeukaryotes. Extensive resource diversity facilitates niche partitioning and promotes cooperative (e.g., cross-feeding) and competitive interactions, resulting in highly complex co-occurrence networks (83). The derived methane and sulfur compounds from cold seep sustain a dynamic community of chemosynthetic prokaryotes (e.g., methanotrophs and sulfide oxidizers), potentially leading to the alpha diversity of prokaryotes in BZ that is comparable to that in EZ and MZ (Fig. 2B). However, the methane and sulfur compounds from the seabed exerted a negligible effect on promoting microeukaryotic diversity, as many microeukaryotes are not chemosynthetic and cannot utilize these compounds for energy. Consequently, the complexity of the microbial network in BZ was lower than that in EZ (Fig. 4B).

The deep-burrowing fauna, such as clams, engage in bioturbation (burrowing activities), which physically mixes sediments, thereby enhancing sediment stability, biogeochemistry, and microbial dynamics (84). Consequently, this physical arrangement facilitates a broad range of microbial metabolic pathways, fostering frequent and diverse species interactions that ultimately lead to a highly connected and complex co-occurrence network (Fig. 6 and Table 3). Furthermore, the clam habitat (R5), frequently representing the initial successional stage of fauna in cold seep environments, is often characterized by fluid dynamics and fluctuating biogeochemical gradients (68–70). These conditions may enhance initial microbial diversities and promote a wide variety of species interactions, leading to the development of complex network structures (Fig. 6 and Table 3). By contrast, the mussel habitat (R1) represents a later successional stage characterized by a high concentration of methane. This environmental condition may shape strong environmental filtering, favoring specialized microbial communities. As a result, the habitat developed a simpler microbial network with fewer species interactions compared with the clam habitat (R5; Fig. 6 and Table 3).

### Shaping factors and mechanisms for microbial community distribution and co-occurrence patterns

In water columns, null model results suggested that deterministic processes primarily influenced the distribution of the microeukaryotic community population, but the prokaryotic counterparts were more closely associated with the stochastic processes. By contrast, deterministic processes appeared to shape the distribution of prokaryotic communities, and stochastic processes were the estimated drivers for microeukaryotic communities in sediments (Fig. 7). In our study, the prokaryotic community distribution was estimated to be predominantly driven by homogenizing dispersal (34.15%, stochastic process) and homogeneous selection (33.83%, deterministic process) in water columns (Fig. 7A). A previous study revealed that stochastic processes predominantly shaped the prokaryotic community distribution, suggesting that the dissemination of prokaryotes throughout interconnected water layers may be largely random. Fluid released from the cold seep may have driven the bacteria upward, reducing dispersal limitation (85). By contrast, homogeneous selection (58%, deterministic process) appeared to be the primary factor influencing the microeukaryotic community distribution in water columns (Fig. 7), characterized by significant correlations between measured environmental factors and microeukaryotic community structures (Fig. 3 and Table 1). Temperature and salinity are important limiting factors in the marine environment, and several studies have identified their effect on microbial community structures (86, 87). Nutrient concentrations are also determinants that influence changes in microbial communities. Inorganic N and P are the bases of biological metabolism (88), and the effects of nutrients on microbial communities may be related to the lack of material inputs in deep-sea environments (89). Furthermore, compared with prokaryotes, deterministic processes exhibited a greater effect on microeukaryotes in water columns. This was reflected by increased microeukaryotic community variation attributed to the measured environmental factors (Fig. 3), as well as their elevated correlation coefficients (Table 1). This may be attributed to prokaryotes' superior metabolic flexibility and reduced susceptibility to environmental factors (90). Homogeneous selection (59%, deterministic process) was identified as the primary deterministic force shaping the prokaryotic community distribution, whereas the microeukaryotic community distribution was estimated to be mainly driven by undominated (59%, stochastic process) and dispersal limitation (32%, stochastic process) in sediments (Fig. 7). Previous studies also showed that horizontal environmental heterogeneity (deterministic process) among different habitats influences microbial community distributions, characterized by several significant correlations between environmental factors and microbial community structures (32, 34). The predominance of stochastic processes in organizing microeukaryotic communities was consistent with a previous study, suggesting that sedimental microeukaryotes are less responsive to environmental variables compared with those in water columns (33). This phenomenon could be attributed to the wide niche diversity of microeukaryotes exhibiting different trophic modes in sediments compared with water columns, such as parasitic (Syndiniales), autotrophic (Ochrophyta), heterotrophic (Ciliophora and MAST-9), and mixotrophic (Retaria) lifestyles (Fig. 4 and Table S3). This diversity may enhance resource (e.g., organic matter) use efficiency, reduce competition, and promote species coexistence. The varying dominance of deterministic and stochastic processes in prokaryotic and microeukaryotic community distribution could be attributed to the large cell sizes, highly complex cell structures, and flagella-aided mobility of microeukaryotes, which enable them to occupy and readily access distinct niches (90).

Our study initially explored the factors and mechanisms shaping species coexistence within microbial communities in the Haima cold seep, revealing that microbial co-occurrence patterns were segregated, and deterministic processes strongly influenced the microbial co-occurrence patterns of EZ and R5 compared with other water layers and habitats, respectively (Fig. 7B). The microbial co-occurrence patterns were segregated, indicating that the microbial co-occurrence patterns were predominantly affected by heterogeneous selection and competitive exclusion (91). The environmental factors exhibited significant correlations with the topological parameters of networks (Fig. 4 and Fig. S3 and S4), indicating that heterogeneous selection played an important role in shaping microbial co-occurrence patterns. The increased deterministic influence on the microbial network in EZ corresponded with the essential function of light-driven primary production in this layer. EZ is characterized by a relatively elevated input of organic carbon and complex biological interactions, creating an environment conducive to niche-based selection. This may result in intensified competition for resources and structured co-occurrence patterns, as communities are influenced by specific environmental conditions. By contrast, MZ and BZ, often oligotrophic and strongly influenced by physical mixing and transport, may primarily be driven by stochastic processes such as homogenizing dispersal and dispersal limitation (92). Moreover, deterministic processes exert a stronger influence on the microbial network in R1 (mussel bed) sediments than in other habitats, which could be attributed to the selection of sedimentary geochemistry and mussels. The high concentration of methane in sediments of mussel habitats may exert a deterministic influence on microbial communities (92). In addition, mussel beds, particularly those involving cold-seep specialists such as *Gigantidas haimaensis*, create highly specialized microenvironments through their biological activities. They form symbiotic relationships with methane-oxidizing bacteria, creating a strong environmental filter that favors a specialized microbial consortium capable of thriving under these conditions (93). Furthermore, the dense aggregation of mussels alters sediment geochemistry and provides a steady substrate, thereby reducing the influence of random colonization and enhancing niche-based selection (94).

### Conclusion

Our study yielded critical insights into the distribution and co-occurrence patterns, as well as the influencing factors and mechanisms of microeukaryotes and prokaryotes in Haima cold seep, by sampling water columns and sediments from various water layers and benthic habitats. Microbial communities showed vertical and horizontal patterns of distribution and co-occurrence in water columns and sediments, respectively. However, their distribution patterns were influenced by varying degrees of significance of ecological processes. The environmental factors and deterministic processes appeared to exert a stronger influence on microeukaryotes than on prokaryotes in the water column, but the reverse was observed in sediments. The prokaryotic intra-domain interactions predominated species correlations, and the bacteria–microeukaryote interactions were more abundant in water columns than in sediments, which indicated that environmental preferences of microorganisms may differ between water columns and sediments. The different species interactions and importance of deterministic and stochastic processes in community distributions between microeukaryotes and prokaryotes, as well as between those in the water column and sediments, were due to their distinct adaptive strategies. In addition, microbial co-occurrence patterns were segregated and likely structured by environmental factors and deterministic processes, such as heterogeneous selection and competitive exclusion. The species interactions were more complex in EZ and clam habitat, and the microbial co-occurrence patterns in EZ and mussel beds were likely driven by selection factors such as light, organic carbon, mussel activities, and high concentrations of methane. Overall, our results indicated that microeukaryotes and prokaryotes employed different strategies to adapt to the environments in deep-sea cold seeps, deepening our understanding of microbial ecology in deep-sea extreme environments.

## Data Availability

The raw sequencing data have been deposited in the NCBI SRA database under the BioProject accession number PRJNA1337497.

## SUPPLEMENTAL MATERIAL

Supplemental figures spectrum.00256-26-s0001.docx Figures S1–S4.

Supplemental tables spectrum.00256-26-s0002.xlsx Tables S1–S7.

ASM does not own the copyrights to Supplemental Material that may be linked to, or accessed through, an article. The authors have granted ASM a non-exclusive, world-wide license to publish the Supplemental Material files. Please contact the corresponding author directly for reuse.

## References

[1] Boetius A, Wenzhöfer F. 2013. Seafloor oxygen consumption fuelled by methane from cold seeps. Nature Geosci 6:725–734. doi:10.1038/ngeo1926

[2] Leprich DJ, Flood BE, Schroedl PR, Ricci E, Marlow JJ, Girguis PR, Bailey JV. 2021. Sulfur bacteria promote dissolution of authigenic carbonates at marine methane seeps. ISME J 15:2043–2056. doi:10.1038/s41396-021-00903-3 33574572 PMC8245480

[3] Joye SB. 2020. The geology and biogeochemistry of hydrocarbon seeps. Annu Rev Earth Planet Sci 48:205–231. doi:10.1146/annurev-earth-063016-020052

[4] Ruff SE, Biddle JF, Teske AP, Knittel K, Boetius A, Ramette A. 2015. Global dispersion and local diversification of the methane seep microbiome. Proc Natl Acad Sci USA 112:4015–4020. doi:10.1073/pnas.1421865112 25775520 PMC4386351

[5] Åström EKL, Carroll ML, Ambrose WG, Sen A, Silyakova A, Carroll J. 2018. Methane cold seeps as biological oases in the high‐Arctic deep sea. Limnol Oceanogr 63:S209–S231. doi:10.1002/lno.10732

[6] Feng JC, Li CR, Tang L, Wu XN, Wang Y, Yang Z, Yuan W, Sun L, Hu W, Zhang S. 2023. Tracing the century-long evolution of microplastics deposition in a cold seep. Adv Sci 10:e2206120. doi:10.1002/advs.202206120 PMC10074074 36737848

[7] Huang Y, Feng J-C, Kong J, Sun L, Zhang M, Huang Y, Tang L, Zhang S, Yang Z. 2023. Community assemblages and species coexistence of prokaryotes controlled by local environmental heterogeneity in a cold seep water column. Sci Total Environ 868:161725. doi:10.1016/j.scitotenv.2023.161725 36669671

[8] Savvichev AS, Kadnikov VV, Kravchishina MD, Galkin SV, Novigatskii AN, Sigalevich PA, Merkel Ay, Ravin NV, Pimenov NV, Flint MV. 2018. Methane as an organic matter source and the trophic basis of a laptev sea cold seep microbial community. Geomicrobiol J 35:411–423. doi:10.1080/01490451.2017.1382612

[9] Dong X, Zhang T, Wu W, Peng Y, Liu X, Han Y, Chen X, Gao Z, Xia J, Shao Z, Greening C. 2024. A vast repertoire of secondary metabolites potentially influences community dynamics and biogeochemical processes in cold seeps. Sci Adv 10:eadl2281. doi:10.1126/sciadv.adl2281 38669328 PMC11051675

[10] Li Z, Pan D, Wei G, Pi W, Zhang C, Wang J-H, Peng Y, Zhang L, Wang Y, Hubert CRJ, Dong X. 2021. Deep sea sediments associated with cold seeps are a subsurface reservoir of viral diversity. ISME J 15:2366–2378. doi:10.1038/s41396-021-00932-y 33649554 PMC8319345

[11] Huang P, Zhao F, Zhou B, Xu K. 2024. Active microeukaryotes hold clues of effects of global warming on benthic diversity and connectivity in the coastal sediments. Ecol Indic 158:111316. doi:10.1016/j.ecolind.2023.111316

[12] Li C, Feng J-C, Chen X, Zhou Y, Liang J, Zhang S. 2024. Behaviours of methane metabolism and community dynamics of methane anaerobic oxidation microbes on carbonate rocks with long-term cultivation in cold seep environment. Appl Energy 365:123296. doi:10.1016/j.apenergy.2024.123296

[13] Yu T, Luo Y, Tan X, Zhao D, Bi X, Li C, Zheng Y, Xiang H, Hu S. 2024. Global marine cold seep metagenomes reveal diversity of taxonomy, metabolic function, and natural products. Genomics Proteomics Bioinformatics 22:qzad006. doi:10.1093/gpbjnl/qzad006 39160620 PMC12016038

[14] Jiang Q, Cao L, Han Y, Li S, Zhao R, Zhang X, Ruff SE, Zhao Z, Peng J, Liao J, Zhu B, Wang M, Lin X, Dong X. 2025. Cold seeps are potential hotspots of deep-sea nitrogen loss driven by microorganisms across 21 phyla. Nat Commun 16:1646. doi:10.1038/s41467-025-56774-1 39952920 PMC11828985

[15] Li WL, Dong X, Lu R, Zhou YL, Zheng PF, Feng D, Wang Y. 2021. Microbial ecology of sulfur cycling near the sulfate-methane transition of deep-sea cold seep sediments. Environ Microbiol 23:6844–6858. doi:10.1111/1462-2920.15796 34622529

[16] Quan Q, Liu J, Xia X, Zhang S, Ke Z, Wang M, Tan Y. 2024. Cold seep nitrogen fixation and its potential relationship with sulfur cycling. Microbiol Spectr 12:e0053624. doi:10.1128/spectrum.00536-24 39171911 PMC11448218

[17] Savvichev AS, Rusanov II, Kadnikov VV, Beletsky AV, Zakcharova EE, Samylina OS, Sigalevich PA, Semiletov IP, Ravin NV, Pimenov NV. 2023. Biogeochemical activity of methane-related microbial communities in bottom sediments of cold seeps of the Laptev sea. Microorganisms 11:250. doi:10.3390/microorganisms11020250 36838215 PMC9964916

[18] Semler AC, Fortney JL, Fulweiler RW, Dekas AE. 2022. Cold seeps on the passive Northern U.S. Atlantic Margin host globally representative members of the seep microbiome with locally dominant strains of archaea. Appl Environ Microbiol 88:e0046822. doi:10.1128/aem.00468-22 35607968 PMC9195954

[19] Wang X, Zhao D, Yu T, Zhu Y, Jiang M, Liu Y, Hu S, Luo Y, Xiang H, Zheng Y. 2025. Biological nitrogen fixation driven by methane anaerobic oxidation supports the complex biological communities in cold-seep habitat. Environ Technol Innov 37:103938. doi:10.1016/j.eti.2024.103938

[20] Pop Ristova P, Wenzhöfer F, Ramette A, Felden J, Boetius A. 2015. Spatial scales of bacterial community diversity at cold seeps (Eastern Mediterranean Sea). ISME J 9:1306–1318. doi:10.1038/ismej.2014.217 25500510 PMC4438319

[21] Shekarriz E, Chen J, Xu Z, Liu H. 2023. Disentangling the functional role of fungi in cold seep sediment. Microbiol Spectr 11:e0197822. doi:10.1128/spectrum.01978-22 36912690 PMC10100914

[22] Zhang X, Wu K, Han Z, Chen Z, Liu Z, Sun Z, Shao L, Zhao Z, Zhou L. 2022. Microbial diversity and biogeochemical cycling potential in deep-sea sediments associated with seamount, trench, and cold seep ecosystems. Front Microbiol 13:1029564. doi:10.3389/fmicb.2022.1029564 36386615 PMC9650238

[23] Li X, Dai Z, Di P, Feng J, Tao J, Chen D, Li N, Li Y. 2021. Distinct bottom-water bacterial communities at methane seeps with various seepage intensities in Haima, South China Sea. Front Mar Sci 8:753952. doi:10.3389/fmars.2021.753952

[24] Zhong S, Feng J, Kong J, Huang Y, Chen X, Zhang S. 2023. Differences in bacterial co-occurrence networks and ecological niches at the surface sediments and bottom seawater in the haima cold seep. Microorganisms 11:3001. doi:10.3390/microorganisms11123001 38138145 PMC10745737

[25] Niu M, Deng L, Su L, Ruff SE, Yang N, Luo M, Qi Q, Li J, Wang F. 2023. Methane supply drives prokaryotic community assembly and networks at cold seeps of the South China Sea. Mol Ecol 32:660–679. doi:10.1111/mec.16786 36408814

[26] Xiong X, Li F, Yang H, Li C, Chen H, He D, Wu QL, Huang S, Ren L. 2025. Seepage area of the cold seep exhibits strong homogeneous selection on prokaryotic community assembly and supports high depth variability of both archaeal and bacterial communities. Microbiol Spectr 13:e0272224. doi:10.1128/spectrum.02722-24 40492763 PMC12210922

[27] Dong D, Li X, Yang M, Gong L, Li Y, Sui J, Gan Z, Kou Q, Xiao N, Zhang J. 2021. Report of epibenthic macrofauna found from haima cold seeps and adjacent deep-sea habitats, South China Sea. Mar Life Sci Technol 3:1–12. doi:10.1007/s42995-020-00073-9 37073389 PMC10077165

[28] Liang J, Feng J-C, Kong J, Huang Y, Zhang H, Zhong S, Tang L, Zhang S. 2023. Microbial communities and mineral assemblages in sediments from various habitats at the haima cold seep, South China Sea. Front Mar Sci 10:1254450. doi:10.3389/fmars.2023.1254450

[29] Xu H, Du M, Li J, Zhang H, Chen W, Wei J, Wu Z, Zhang H, Li J, Chen S, Ta K, Bai S, Peng X. 2020. Spatial distribution of seepages and associated biological communities within haima cold seep field, South China Sea. J Sea Res 165:101957. doi:10.1016/j.seares.2020.101957

[30] Cui H, Su X, Chen F, Holland M, Yang S, Liang J, Su P, Dong H, Hou W. 2019. Microbial diversity of two cold seep systems in gas hydrate-bearing sediments in the South China Sea. Mar Environ Res 144:230–239. doi:10.1016/j.marenvres.2019.01.009 30732863

[31] Dong X, Peng Y, Wang M, Woods L, Wu W, Wang Y, Xiao X, Li J, Jia K, Greening C. 2023. Evolutionary ecology of microbial populations inhabiting deep sea sediments associated with cold seeps. Nat Commun 14:1127. doi:10.1038/s41467-023-36877-3 36854684 PMC9974965

[32] Xu Z, Chen J, Li Y, Shekarriz E, Wu W, Chen B, Liu H. 2023. High microeukaryotic diversity in the cold-seep sediment. Microb Ecol 86:2003–2020. doi:10.1007/s00248-023-02212-y 36973438

[33] Xu Z, Chen J, Liang W, Chen ZL, Wu W, Xia X, Chen B, He D, Liu H. 2025. Contrasting diversity patterns between microeukaryotic and prokaryotic communities in cold-seep sediments. ISME Commun 5:ycaf002. doi:10.1093/ismeco/ycaf002 40041702 PMC11879339

[34] Wu Q, Feng J, Huang Y, Zhong S, Li C, Zhang S. 2025. Heterogeneity of the horizontal environment drives community assemblages and species coexistence of prokaryotic communities in cold seep sediments. Front Microbiol 16:1–40. doi:10.3389/fmicb.2025.1687453 PMC12675211 41356487

[35] Arístegui J, Gasol JM, Duarte CM, Herndld GJ. 2009. Microbial oceanography of the dark ocean’s pelagic realm. Limnol Oceanogr 54:1501–1529. doi:10.4319/lo.2009.54.5.1501

[36] Giner CR, Pernice MC, Balagué V, Duarte CM, Gasol JM, Logares R, Massana R. 2020. Marked changes in diversity and relative activity of picoeukaryotes with depth in the world ocean. ISME J 14:437–449. doi:10.1038/s41396-019-0506-9 31645670 PMC6976695

[37] Walters W, Hyde ER, Berg-Lyons D, Ackermann G, Humphrey G, Parada A, Gilbert JA, Jansson JK, Caporaso JG, Fuhrman JA, Apprill A, Knight R. 2016. Improved bacterial 16S rRNA Gene (V4 and V4-5) and fungal internal transcribed spacer marker gene primers for microbial community surveys. mSystems 1:e00009-15. doi:10.1128/mSystems.00009-15 PMC5069754 27822518

[38] Stoeck T, Behnke A, Christen R, Amaral-Zettler L, Rodriguez-Mora MJ, Chistoserdov A, Orsi W, Edgcomb VP. 2009. Massively parallel tag sequencing reveals the complexity of anaerobic marine protistan communities. BMC Biol 7:72. doi:10.1186/1741-7007-7-72 19886985 PMC2777867

[39] Callahan BJ, McMurdie PJ, Rosen MJ, Han AW, Johnson AJA, Holmes SP. 2016. DADA2: high-resolution sample inference from Illumina amplicon data. Nat Methods 13:581–583. doi:10.1038/nmeth.3869 27214047 PMC4927377

[40] Bolyen E, Rideout JR, Dillon MR, Bokulich NA, Abnet CC, Al-Ghalith GA, Alexander H, Alm EJ, Arumugam M, Asnicar F, et al.. 2019. Reproducible, interactive, scalable and extensible microbiome data science using QIIME 2. Nat Biotechnol 37:852–857. doi:10.1038/s41587-019-0209-9 31341288 PMC7015180

[41] Caporaso JG, Kuczynski J, Stombaugh J, Bittinger K, Bushman FD, Costello EK, Fierer N, Peña AG, Goodrich JK, Gordon JI, et al.. 2010. QIIME allows analysis of high-throughput community sequencing data. Nat Methods 7:335–336. doi:10.1038/nmeth.f.303 20383131 PMC3156573

[42] Quast C, Pruesse E, Yilmaz P, Gerken J, Schweer T, Yarza P, Peplies J, Glöckner FO. 2012. The silva ribosomal RNA gene database project: improved data processing and web-based tools. Nucleic Acids Res 41:D590–D596. doi:10.1093/nar/gks1219 23193283 PMC3531112

[43] Shannon CE. 1948. A mathematical theory of communication. Bell System Technical Journal 27:379–423. doi:10.1002/j.1538-7305.1948.tb01338.x

[44] Pielou EC. 1966. The measurement of diversity in different types of biological collections. J Theor Biol 13:131–144. doi:10.1016/0022-5193(66)90013-0

[45] Gehan EA. 1965. A generalized Wilcoxon test for comparing arbitrarily singly-censored samples. Biometrika 52:203–224. doi:10.1093/biomet/52.1-2.203 14341275

[46] Breslow N. 1970. A generalized Kruskal-Wallis test for comparing K samples subject to unequal patterns of censorship. Biometrika 57:579–594. doi:10.1093/biomet/57.3.579

[47] Beals EW. 1984. Bray-Curtis ordination: an effective strategy for analysis of multivariate ecological data, p 1–55. In MacFadyen A, Ford ED (ed), Advances in ecological research. Elsevier, Amsterdam, Netherlands.

[48] Clarke KR, Somerfield PJ, Gorley RN. 2008. Testing of null hypotheses in exploratory community analyses: similarity profiles and biota-environment linkage. J Exp Mar Biol Ecol 366:56–69. doi:10.1016/j.jembe.2008.07.009

[49] Team RC. 2025. R: a language and environment for statistical computing. Vienna, Austria. https://www.R-project.org.

[50] Blanchet FG, Friendly M, Kindt R, McGlinn D, Minchin PR, O’Hara R, Solymos P, H. Stevens M, Eduard S, Helene W. 2013. Vegan: community ecology package version 2.0-10. Available from: https://CRANR-projectorg/package=vegan

[51] Etten EV. 2005. Multivariate analysis of ecological data Using canoco. Austral Ecol 30:486–487. doi:10.1111/j.1442-9993.2005.01433.x

[52] Manly BF. 2018. Randomization, bootstrap and monte carlo methods in biology. CRC press, New York, USA. Available from: 10.1201/9781315273075

[53] Harrell FE, Dupont C. 2020. Hmisc: harrell miscellaneous, r package version 4.0. Available from: https://harrelfe.r-universe.dev/Hmisc

[54] Csardi G. 2021. Package igraph. Available from: https://igraph.org

[55] Bastian M, Heymann S, Jacomy M. 2009 Gephi: an open source software for exploring and manipulating networks. ICWSM 3:361–362. doi:10.1609/icwsm.v3i1.13937

[56] Erdős P, Rényi A. 1960. On the evolution of random graphs. Publ Math Inst Hung Acad Sci 5:17–61.

[57] Guimerà R, Nunes Amaral LA. 2005. Functional cartography of complex metabolic networks. Nature 433:895–900. doi:10.1038/nature03288 15729348 PMC2175124

[58] Strogatz SH. 2001. Exploring complex networks. Nature 410:268–276. doi:10.1038/35065725 11258382

[59] Yuan MM, Guo X, Wu L, Zhang Y, Xiao N, Ning D, Shi Z, Zhou X, Wu L, Yang Y, Tiedje JM, Zhou J. 2021. Climate warming enhances microbial network complexity and stability. Nat Clim Chang 11:343–348. doi:10.1038/s41558-021-00989-9

[60] Kembel SW, Cowan PD, Helmus MR, Cornwell WK, Morlon H, Ackerly DD, Blomberg SP, Webb CO. 2010. Picante: r tools for integrating phylogenies and ecology. Bioinformatics 26:1463–1464. doi:10.1093/bioinformatics/btq166 20395285

[61] Stegen JC, Lin X, Fredrickson JK, Chen X, Kennedy DW, Murray CJ, Rockhold ML, Konopka A. 2013. Quantifying community assembly processes and identifying features that impose them. ISME J 7:2069–2079. doi:10.1038/ismej.2013.93 23739053 PMC3806266

[62] Stegen JC, Lin X, Konopka AE, Fredrickson JK. 2012. Stochastic and deterministic assembly processes in subsurface microbial communities. ISME J 6:1653–1664. doi:10.1038/ismej.2012.22 22456445 PMC3498916

[63] Chase JM, Kraft NJB, Smith KG, Vellend M, Inouye BD. 2011. Using null models to disentangle variation in community dissimilarity from variation in α-diversity. Ecosphere 2:1-11. doi:10.1890/ES10-00117.1

[64] Stone L, Roberts A. 1990. The checkerboard score and species distributions. Oecologia 85:74–79. doi:10.1007/BF00317345 28310957

[65] Crump BC, Peterson BJ, Raymond PA, Amon RMW, Rinehart A, McClelland JW, Holmes RM. 2009. Circumpolar synchrony in big river bacterioplankton. Proc Natl Acad Sci USA 106:21208–21212. doi:10.1073/pnas.0906149106 19940248 PMC2783008

[66] Gotelli N, Hart E, Ellison A, Hart ME. 2015. EcoSimR: null model analysis for ecological data. 10.5281/zenodo.16522.

[67] Boltovskoy D. 2017. Vertical distribution patterns of *Radiolaria Polycystina* (Protista) in the world ocean: living ranges, isothermal submersion and settling shells. J Plankton Res 39:330–349. doi:10.1093/plankt/fbx003

[68] Cordes EE, Bergquist DC, Fisher CR. 2009. Macro-ecology of gulf of mexico cold seeps. Annu Rev Mar Sci 1:143–168. doi:10.1146/annurev.marine.010908.163912 21141033

[69] Bowden DA, Rowden AA, Thurber AR, Baco AR, Levin LA, Smith CR. 2013. Cold seep epifaunal communities on the hikurangi margin, New Zealand: composition, succession, and vulnerability to human activities. PLoS One 8:e76869. doi:10.1371/journal.pone.0076869 24204691 PMC3800081

[70] Chen Y, Li J, Li N, Di P, Li Q, Tao J, Zhang S. 2025. All roads lead to Rome: multiple trajectories of faunal succession in deep-sea cold seeps. Sci Bull Sci Found Philipp 70:871–875. doi:10.1016/j.scib.2024.12.011 39710521

[71] Chen Y, Dai T, Li N, Li Q, Lyu Y, Di P, Lyu L, Zhang S, Li J. 2023. Environmental heterogeneity shapes the C and S cycling-associated microbial community in Haima's cold seeps. Front Microbiol 14:1199853. doi:10.3389/fmicb.2023.1199853 37502402 PMC10370420

[72] Pande S, Kost C. 2017. Bacterial unculturability and the formation of intercellular metabolic networks. Trends Microbiol 25:349–361. doi:10.1016/j.tim.2017.02.015 28389039

[73] Liu S, Hu R, Strong PJ, Saleem M, Zhou Z, Luo Z, Wu Y, He Z, Wang C. 2023. Vertical connectivity of microbiome and metabolome reveals depth-dependent variations across a deep cold-seep water column. Environ Res 239:117310. doi:10.1016/j.envres.2023.117310 37805181

[74] Quan Q, Liu J, Li C, Ke Z, Tan Y. 2024. Insights into prokaryotic communities and their potential functions in biogeochemical cycles in cold seep. mSphere 9:e00549-24. doi:10.1128/msphere.00549-24 39269181 PMC11524163

[75] Countway PD, Gast RJ, Dennett MR, Savai P, Rose JM, Caron DA. 2007. Distinct protistan assemblages characterize the euphotic zone and deep sea (2500 m) of the western North Atlantic (Sargasso Sea and Gulf Stream). Environ Microbiol 9:1219–1232. doi:10.1111/j.1462-2920.2007.01243.x 17472636

[76] Schnetzer A, Moorthi SD, Countway PD, Gast RJ, Gilg IC, Caron DA. 2011. Depth matters: microbial eukaryote diversity and community structure in the eastern North Pacific revealed through environmental gene libraries. Deep Sea Research Part I: Oceanographic Research Papers 58:16–26. doi:10.1016/j.dsr.2010.10.003

[77] Bråte J, Krabberød AK, Dolven JK, Ose RF, Kristensen T, Bjørklund KR, Shalchian-Tabrizi K. 2012. Radiolaria associated with large diversity of marine alveolates. Protist 163:767–777. doi:10.1016/j.protis.2012.04.004 22658831

[78] Zhang Y, Huang N, Wang M, Liu H, Jing H. 2021. Microbial eukaryotes associated with sediments in deep-sea cold seeps. Front Microbiol 12:782004. doi:10.3389/fmicb.2021.782004 35003010 PMC8740301

[79] Jiang Q, Jing H, Li X, Wan Y, Chou I-M, Hou L, Dong H, Niu Y, Gao D. 2023. Active pathways of anaerobic methane oxidization in deep-sea cold seeps of the South China Sea. Microbiol Spectr 11:e0250523. doi:10.1128/spectrum.02505-23 37916811 PMC10715046

[80] Peng C, Jiang H, Huang L, Hou W, Yang J, Wang S, Huang Q, Deng S, Dong H. 2013. Abundance and diversity of ammonia-oxidizing bacteria and archaea in cold springs on the Qinghai-Tibet Plateau. Geomicrobiol J 30:530–539. doi:10.1080/01490451.2012.737089

[81] Wu Y, Qiu J-W, Qian P-Y, Wang Y. 2018. The vertical distribution of prokaryotes in the surface sediment of Jiaolong cold seep at the northern South China Sea. Extremophiles 22:499–510. doi:10.1007/s00792-018-1012-0 29442249

[82] Li Y, Ye Z, Lai M-C, Liu C-S, Paull CK, Lin S, Lai S-J, You Y-T, Wu S-Y, Hung C-C, Ding J-Y, Shih C-J, Wu Y-C, Zhao J, Xiao W, Wu C-H, Dong G, Zhang H, Qiu W, Wang S, Chen S-C. 2024. Microbial communities in and around the siboglinid tubeworms from the South Yungan East Ridge cold seep offshore Southwestern Taiwan at the Northern South China sea. Microorganisms 12:2452. doi:10.3390/microorganisms12122452 39770655 PMC11676240

[83] Lima-Mendez G, Faust K, Henry N, Decelle J, Colin S, Carcillo F, Chaffron S, Ignacio-Espinosa JC, Roux S, Vincent F, et al.. 2015. Determinants of community structure in the global plankton interactome. Science 348:1262073. doi:10.1126/science.1262073 25999517

[84] Morelle J, Huguet A, Richard A, Laverman AM, Roose-Amsaleg C, Parlanti E, Sourzac M, Mesnage V, Lecoq N, Deloffre J, Viollier E, Maire O, Orvain F. 2024. Antagonistic impacts of benthic bioturbator species: interconnected effects on sedimentary properties, biogeochemical variables, and microbial dynamics. J Exp Mar Biol Ecol 573:152000. doi:10.1016/j.jembe.2024.152000

[85] Lyu Y, Zhang J, Chen Y, Li Q, Ke Z, Zhang S, Li J. 2023. Distinct diversity patterns and assembly mechanisms of prokaryotic microbial sub-community in the water column of deep-sea cold seeps. J Environ Manage 348:119240. doi:10.1016/j.jenvman.2023.119240 37837767

[86] Zhu C, Liu W, Li X, Xu Y, El‐Serehy HA, Al‐Farraj SA, Ma H, Stoeck T, Yi Z. 2021. High salinity gradients and intermediate spatial scales shaped similar biogeographical and co‐occurrence patterns of microeukaryotes in a tropical freshwater‐saltwater ecosystem. Environ Microbiol 23:4778–4796. doi:10.1111/1462-2920.15668 34258839

[87] Zhu C, Wang C, Han J, Quan Z, Zhang J, Lei X, Zhang R, Yi Z. 2025. Temporal factors and habitats drive the variation of microbial distributions and co-occurrence patterns in the Pearl River Estuary. BMC Microbiol 25:609. doi:10.1186/s12866-025-04239-2 41039236 PMC12492964

[88] Wang L, Zhang J, Li H, Yang H, Peng C, Peng Z, Lu L. 2018. Shift in the microbial community composition of surface water and sediment along an urban river. Sci Total Environ 627:600–612. doi:10.1016/j.scitotenv.2018.01.203 29426184

[89] Orcutt BN, Sylvan JB, Knab NJ, Edwards KJ. 2011. Microbial ecology of the dark ocean above, at, and below the seafloor. Microbiol Mol Biol Rev 75:361–422. doi:10.1128/MMBR.00039-10 21646433 PMC3122624

[90] Wan W, Grossart HP, He D, Liu W, Wang S, Yang Y. 2023. Differentiation strategies for planktonic bacteria and eukaryotes in response to aggravated algal blooms in urban lakes. iMeta 2:e84. doi:10.1002/imt2.84 38868338 PMC10989909

[91] Santillan E, Seshan H, Constancias F, Drautz-Moses DI, Wuertz S. 2019 Frequency of disturbance alters diversity, function, and underlying assembly mechanisms of complex bacterial communities. npj Biofilms Microbiomes 5. doi:10.1038/s41522-019-0079-4 PMC6370796 30774969

[92] Wang Q, Wang C, Chen X, Shen Z, Gong Y, Hu Y, Gao G, Shao K, Tang X. 2025. Pico- and nano-eukaryote community assembly in salinity-gradient lakes: diversity patterns, ecological network and environmental correlates. Environ Res 283:122199. doi:10.1016/j.envres.2025.122199 40541901

[93] Sun L, Liu X, Zhou L, Wang H, Lian C, Zhong Z, Wang M, Chen H, Li C. 2025. Shallow-water mussels (*Mytilus galloprovincialis*) adapt to deep-sea environment through transcriptomic and metagenomic insights. Commun Biol 8:46. doi:10.1038/s42003-024-07382-0 39806046 PMC11729891

[94] Guan H, Kiel S, Xu X, Fan J, Zhang Y, Miao X, Nan H, Wu N, Li S. 2026. Sterols transferred from soft tissues to bivalve shells: a new tracer of molecular paleontology. Geology 54:111–116. doi:10.1130/G53933.1

