# Supplementary material for: The patterns of microbial community distribution and co-occurrence in water columns and sediments of Haima cold seep
Source: Microbiol Spectr. 2026 Jun 11;14(7):e00256-26. doi: 10.1128/spectrum.00256-26 (PMC13339816; doi:10.1128/spectrum.00256-26)
Supplement: Supplemental figures — Figures S1–S4. [file spectrum.00256-26-s0001.docx]

SUPPLEMENTARY FIGURES


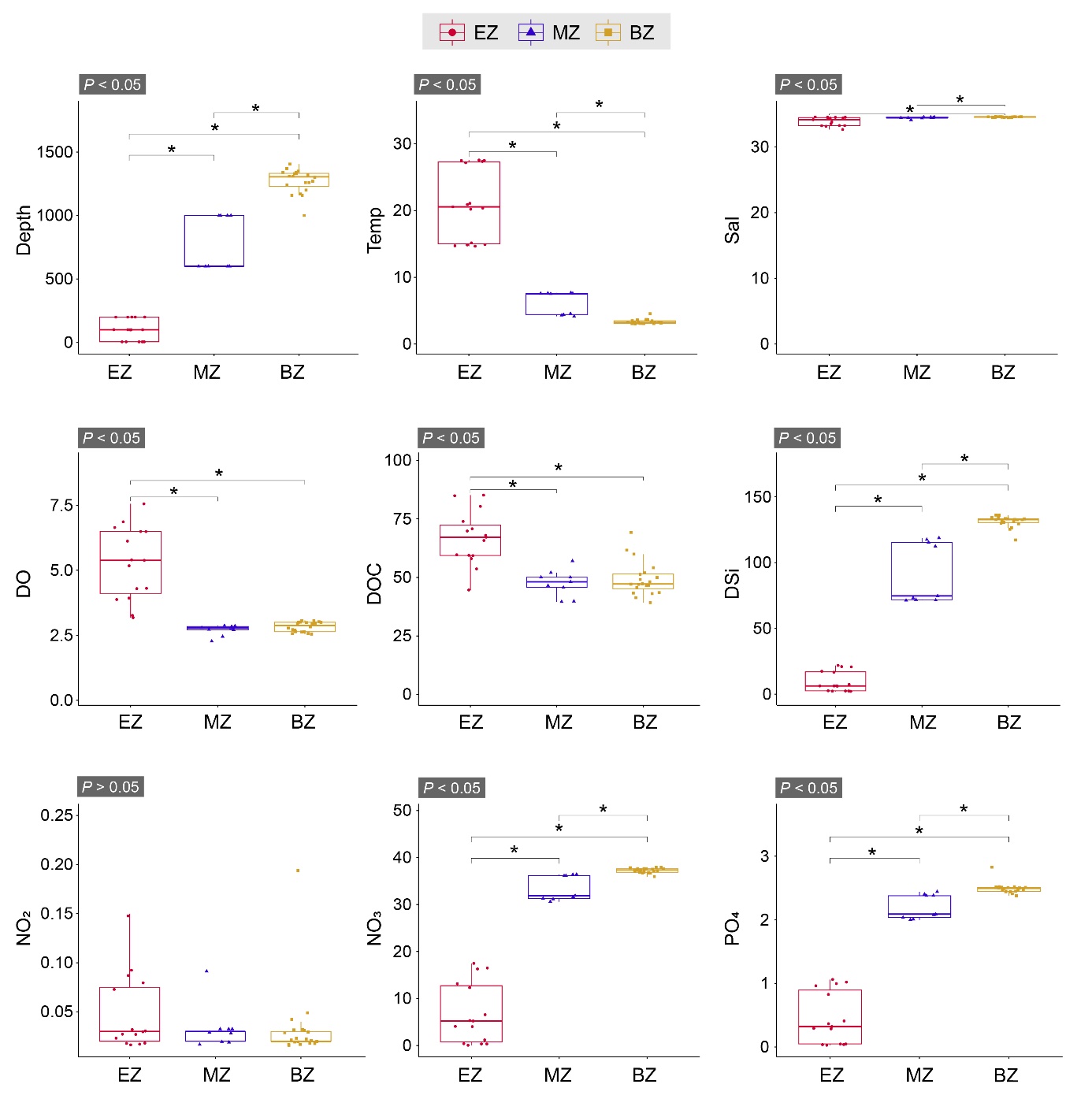


**FIG S1** Environmental factors of the water column in the Haima cold seep. “*” represents significant difference between groups (*P* < 0.05). Abbreviations: Temp, temperature; Sal, salinity; DO, dissolved oxygen; DOC, dissolved organic carbon; DSi, dissolved silicate; NO_2_^-^, nitrite nitrogen; NO_3_^-^, nitrate nitrogen; PO_4_^3-^, phosphate phosphorus.


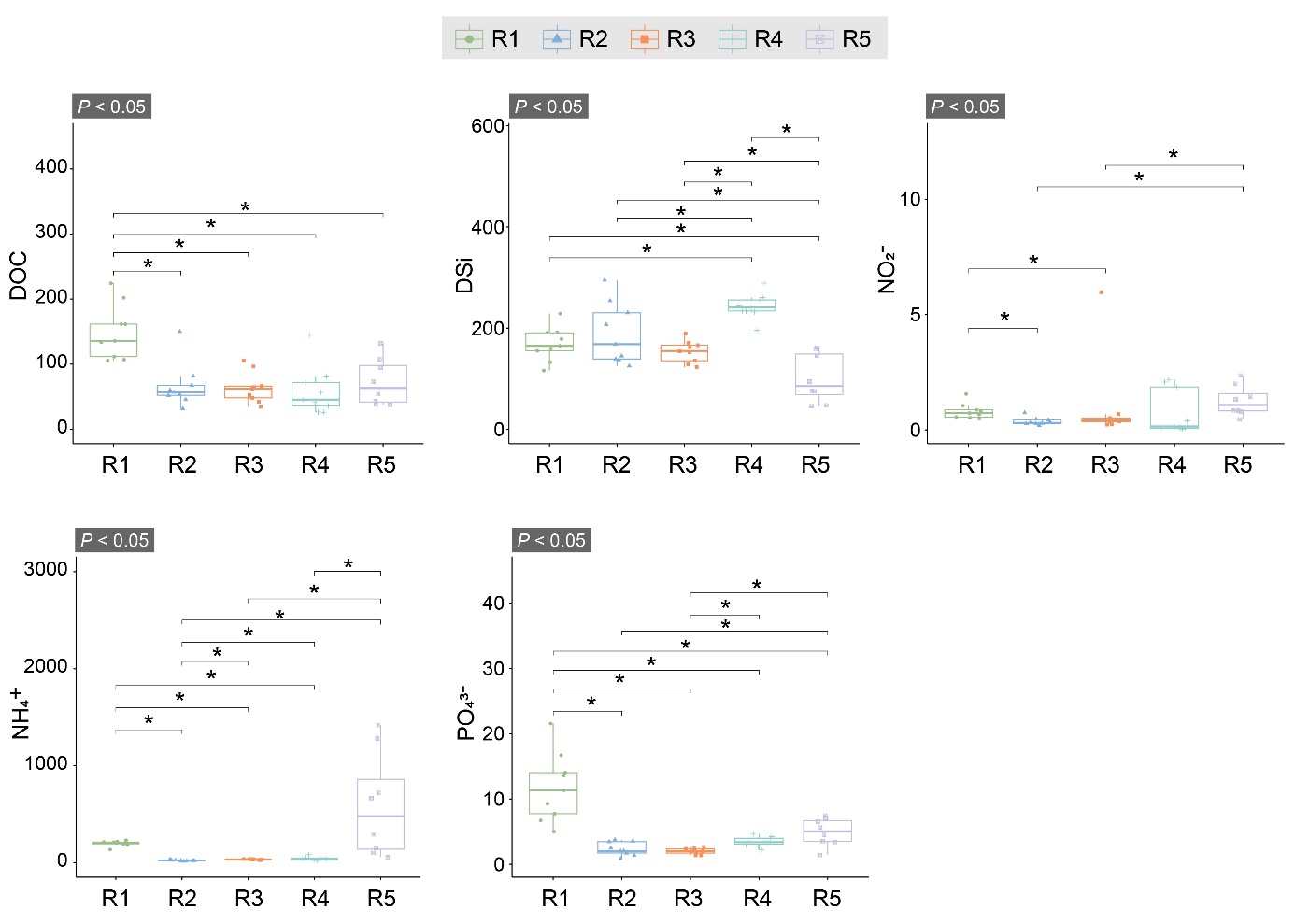


**FIG S2** Environmental factors of the sediments across different bottom habitat in the Haima cold seep. “*” means significant difference between groups (*P* < 0.05). Abbreviations: DOC, dissolved organic carbon; DSi, dissolved silicate; NO_2_^-^, nitrite nitrogen; NO_3_^-^, nitrate nitrogen; PO_4_^3-^, phosphate phosphorus.


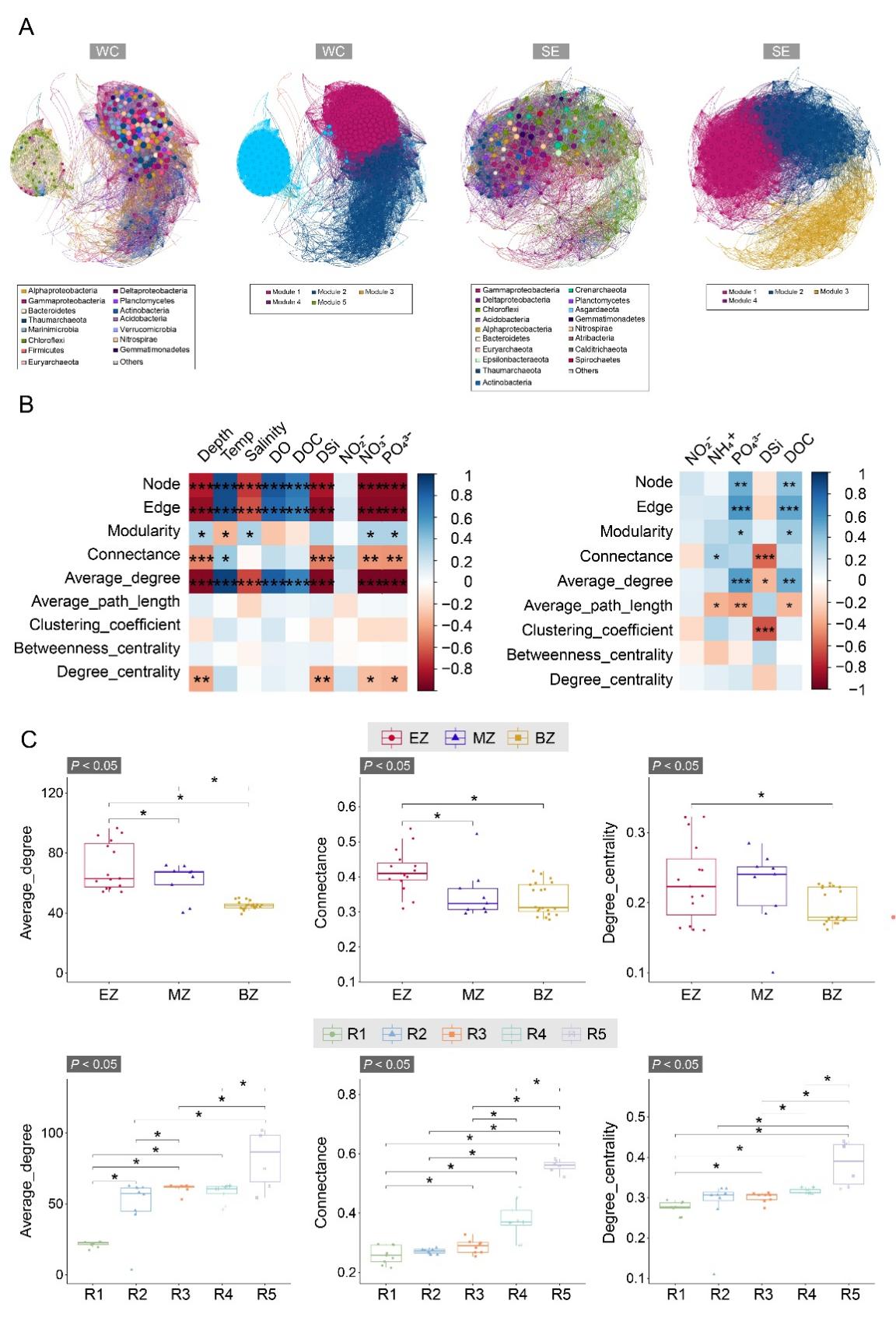


**FIG S3** Co-occurrence patterns of prokaryotic communities from the water columns and sediments based on network analysis. Nodes are coloured according to different taxonomic groups, modules and domains, respectively **(A)**. Spearman correlations between the topological features and environmental factors **(B)**. Significance levels are denoted with **P* < 0.05; ***P* < 0.01; ****P* < 0.001. Comparison of node-level topological features among co-occurrence networks from the three water layers and five bottom habitats **(C)**. Abbreviations: WC, water column samples; SE, sediment samples.


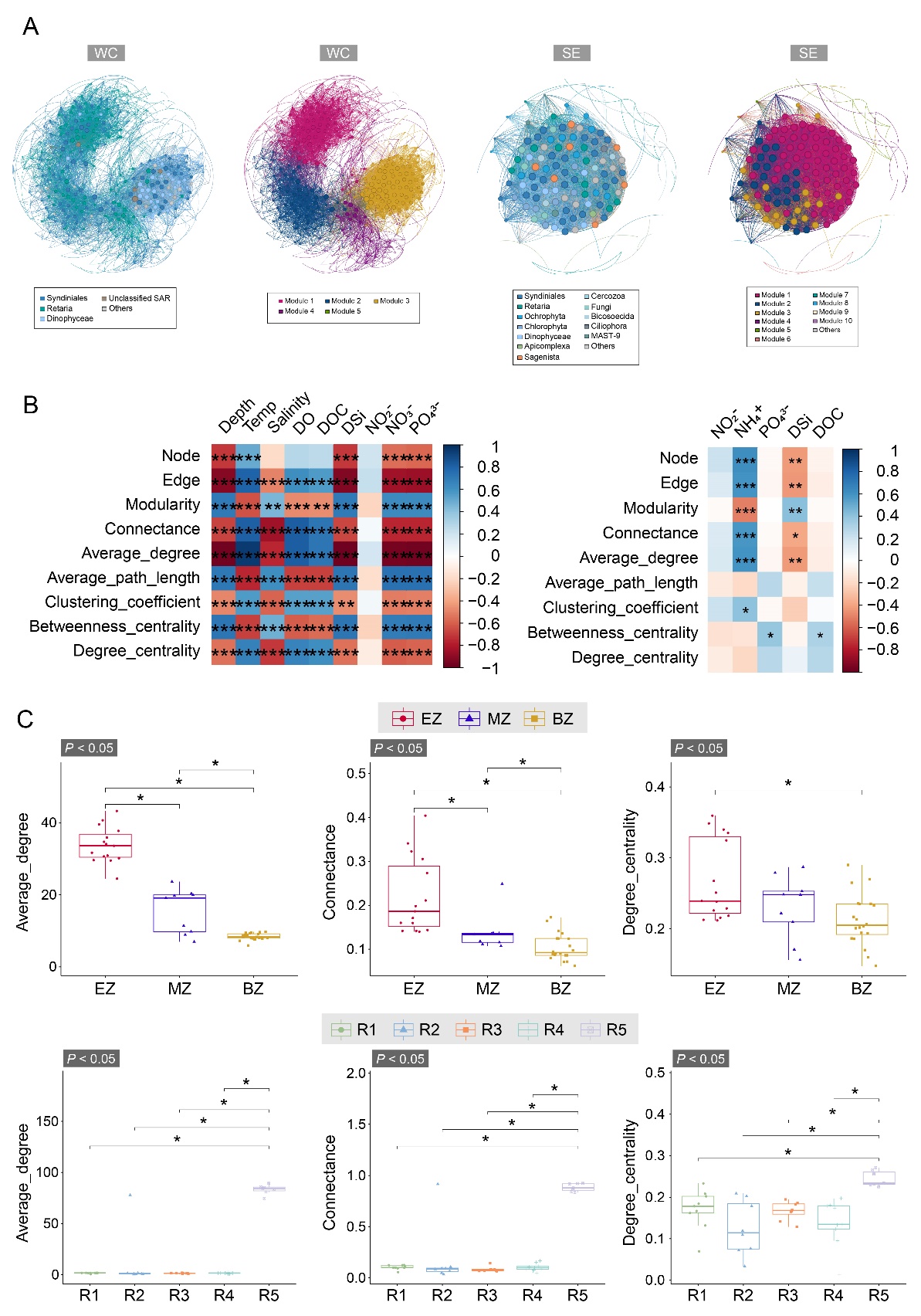


**FIG S4** Co-occurrence patterns of microeukaryotic communities from the water columns and sediments based on network analysis. Nodes are coloured according to different taxonomic groups, modules and domains, respectively **(A)**. Spearman correlations between the topological features and environmental factors **(B)**. Significance levels are denoted with **P* < 0.05; ***P* < 0.01; ****P* < 0.001. Comparison of node-level topological features among co-occurrence networks from the three water layers and five bottom habitats **(C)**. Abbreviations: WC, water column samples; SE, sediment samples.
